# Caspase-11 signaling enhances graft-versus-host disease

**DOI:** 10.1038/s41467-019-11895-2

**Published:** 2019-09-06

**Authors:** Yanyan Lu, Ran Meng, Xiangyu Wang, Yajing Xu, Yiting Tang, Jianfeng Wu, Qianqian Xue, Songlin Yu, Mingwu Duan, Dongyong Shan, Qingde Wang, Haichao Wang, Timothy R. Billiar, Xianzhong Xiao, Fangping Chen, Ben Lu

**Affiliations:** 1 Department of Hematology and Critical Care Medicine, The 3rd Xiangya Hospital, Central South University, Changsha, 410000 P.R. China; 2 Department of Hematology, Xiangya Hospital, Central South University, Changsha, 410008 P.R. China; 3 Department of Physiology, School of Basic Medical Science, Central South University, Changsha, Hunan Province 410000 P.R. China; 4 State Key Laboratory of Cellular Stress Biology Innovation Center for Cell Signaling Network School of Life Sciences, Xiamen University, Xiamen, Fujian Province 361005 P.R. China; 5 The Feinstein Institute for Medical Research, Northwell Health, 350 Community Drive, Manhasset, NY 11030 USA; 6 Department of Surgery, University of Pittsburgh Medical Center, Pittsburgh, PA 15213 USA; 7 Key Laboratory of Sepsis Translational Medicine of Hunan, Central South University, Changsha, Hunan Province 410000 P.R. China; 8 Department of Pathophysiology, School of Basic Medical Science, Central South University, Changsha, Hunan Province 410000 P.R. China

**Keywords:** Cancer immunotherapy, Bone marrow transplantation, Cell death and immune response, Inflammasome, Allotransplantation

## Abstract

Acute graft-versus-host disease (GVHD) remains a major obstacle for the wider usage of allogeneic hematopoietic stem cell transplantation (allo-HSCT), which is an effective therapy for hematopoietic malignancy. Here we show that caspase-11, the cytosolic receptor for bacterial endotoxin (lipopolysaccharide: LPS), enhances GVHD severity. Allo-HSCT markedly increases the LPS-caspase-11 interaction, leading to the cleavage of gasdermin D (GSDMD). Caspase-11 and GSDMD mediate the release of interleukin-1α (IL-1α) in allo-HSCT. Deletion of *Caspase-11* or *Gsdmd*, inhibition of LPS-caspase-11 interaction, or neutralizing IL-1α uniformly reduces intestinal inflammation, tissue damage, donor T cell expansion and mortality in allo-HSCT. Importantly, *Caspase-11* deficiency does not decrease the graft-versus-leukemia (GVL) activity, which is essential to prevent cancer relapse. These findings have major implications for allo-HSCT, as pharmacological interference with the caspase-11 signaling might reduce GVHD while preserving GVL activity.

## Abstract (web summary)

An increasing number of inflammatory pathologies is associated with IL-1 production downstream of caspases 1 and 11. Here the authors show that graft-versus-host-disease (GvHD) is diminished in mice with genetic or pharmacological ablation of caspase-11, and provide mechanistic insights into the signals leading to caspase-11 activation in GvHD.

## Introduction

The success of allogeneic hematopoietic stem cell transplantation (allo-HSCT), a standard therapy for conditions such as hematopoietic malignancies and inherited hematopoietic disorders, is limited by the mortality and morbility associated with graft-versus-host disease (GVHD)^1,2^. In allo-HSCT, irradiation- or chemotherapy-induced gastrointestinal (GI) damage allows the translocation of bacteria or lipopolysaccharide (LPS), the major cell-wall component of Gram-negative bacteria, from GI tract into the systemic circulation^1,3^. Serum and tissue levels of LPS are closely correlated with GVHD severity and mortality^3,4^. LPS antagonism reduces GVHD while preserving graft-versus-leukemia (GVL) activity in experimental models^5^. Further, antibodies against LPS or Gram-negative bacteria significantly reduces the incidence of GVHD in clinical trials^6,7^. It has been proposed that LPS or Gram-negative bacteria enhance GVHD through toll-like receptor 4 (TLR4), a well-established cell surface LPS receptor. However, allo-HSCT recipient mice that express a signaling-deficient *Tlr4* mutant developed fulminant GVHD and increased intestinal damage compared to their wild-type (WT) counterparts^8^. These observations raise a possibility that LPS or Gram-negative bacteria might enhance GVHD through TLR4-independent signaling pathways.

Caspase-11 is a cytosolic LPS receptor that senses various Gram-negative bacteria infections^9–15^. Upon activation by intracellular LPS, caspase-11 oligomerizes into protein complexes and enzymatically cleaves gasdermin D (GSDMD) into pore-forming peptides, leading to a lytic form of cell death, termed pyroptosis^16–18^. This process destroys the intracellular niche for microbes and triggers inflammation by releasing alarmins, such as interleukin-1α (IL-1α)^12,13,15^. Loss of caspase-11 renders mice susceptible to *Burkholderia pseudomallei*, a Gram-negative bacterium endemic to Southeast Asia that causes melioidosis^12^. However, over-activation of caspase-11 in endotoxemia or polymicrobial sepsis leads to organ injury and lethality^10,19–22^. Dysregulated activation of caspase-11 also contributes to the pathogenesis of age-related macular degeneration^23^. Based on these findings and the demonstrated role of LPS in the development of GVHD, we hypothesize that caspase-11 signaling contributes to the pathogenesis of GVHD in allo-HSCT.

In this study, we show that the LPS–caspase-11 interaction is important for the development of GVHD in allo-HSCT. Recognition of LPS by caspase-11 leads to the cleavage of GSDMD, which is essential for the release of IL-1α after bone marrow transplantation (BM). Disruption of the LPS–caspase-11 interaction, deletion of *Caspase-11* or *Gsdmd*, or neutralization of IL-1α uniformly reduces intestinal inflammation, donor T cell expansion, and mortality in allo-HSCT. Moreover, *Caspase-11* deficiency does not compromise the GVL effect. These findings suggest that pharmacological inhibition of the caspase-11 signaling might reduce GVHD while preserving GVL activity in allo-HSCT.

## Results

### Caspase-11 enhances GVHD in allo-HSCT

**Fig. 1:**
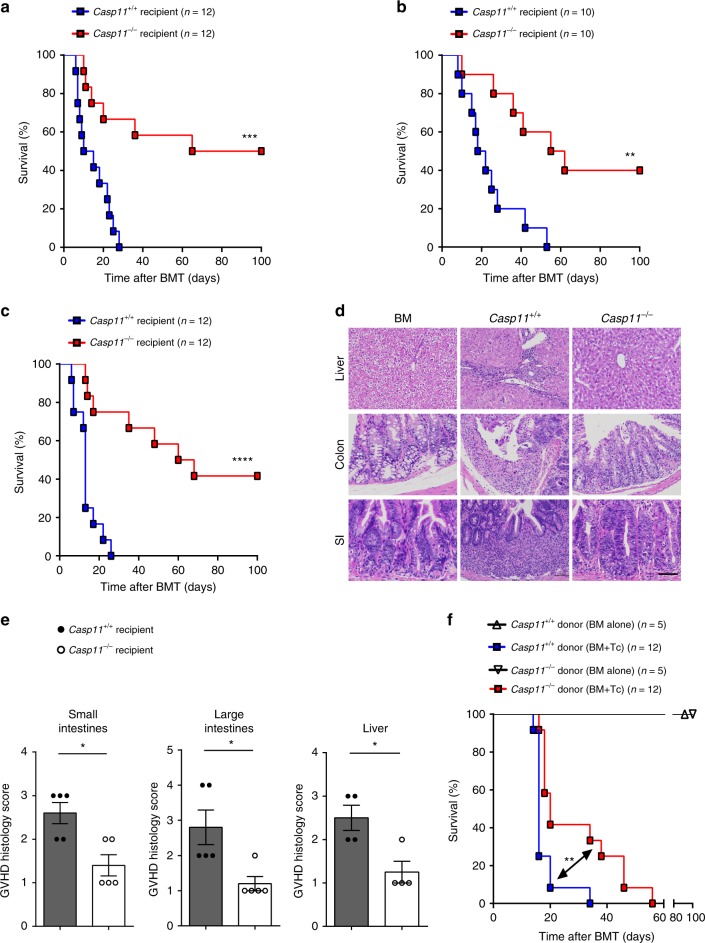
Caspase-11 (Casp11) enhances graft-versus-host disease (GVHD) in allogeneic hematopoietic stem cell transplantation (allo-HSCT). **a** Percentage survival of *Casp11*^+/+^ recipient mice versus *Casp11*^−/−^ recipient mice (BALB/c → C57BL/6 combination) receiving BALB/c bone marrow (BM) and T cells is shown (*n* = 12 in each group). (***P = 0.0008; differences in animal survival were analyzed by log-rank test). **b** Survival of *Casp11*^+/+^ and *Casp11*^−/−^ (Jax) recipients receiving allo-HSCT from BALB/c donors after TBI (total body irradiation) based conditioning is shown (*n* = 10 in each group) (**P = 0.0022; differences in animal survival were analyzed by log-rank test). **c** Survival of *Casp11*^+/+^ recipient mice versus *Casp11*^−/−^ recipient mice receiving allo-HSCT from BALB/c donors after chemotherapy (busulfan/cyclophosphamide (BU/CY))-based conditioning is shown (*n* = 12 in each group). The experiment was performed twice and data were pooled. (****P < 0.0001, differences in animal survival were analyzed by log-rank test). **d** Photomicrographs depicting the average disease score morphology from one representative experiment out of two separate experiments are depicted. Scale bar: 100 μm. BM represents C57BL/6 recipients receiving BALB/c BM group. *Casp11*^+/+^ and *Casp11*^−/−^ recipients represent mice receiving BALB/c BM and T cells group. **e** Histological analysis of the small intestines, large intestines, and liver from *Casp11*^+/+^ and *Casp11*^−/−^ recipient mice receiving BALB/c BM and T cells for GVHD severity on day 14. Data are presented as mean ± SEM. *P < 0.05. An unpaired Student’s *t* test (two-sided) was used. **f** Survival of wild-type (WT) recipient mice (C57BL/6 → BALB/c combination) receiving *Casp11*^+/+^ versus *Casp11*^−/−^ BM with or without T cells is shown. The experiment was performed twice and the results were pooled. *Casp11*^+/+^ versus *Casp11*^−/−^ (**P = 0.0054; differences in animal survival were analyzed by log-rank test)

To determine the role of caspase-11 in GVHD following allo-HSCT, T cells purified from WT mice (on a BALB/c background, H2d) were transplanted together with BM into lethally irradiated major histocompatibility complex (MHC)-mismatched WT or *Caspase-11*-deficient recipient mice (on a B6 background, H2b, provided by the University of Pittsburgh). We observed that genetic deletion of *Caspase-11* in recipients significantly improved survival (Fig. 1a). These findings were reproduced when we used a different strain of *Caspase-11*-deficient mice (on a B6 background, from the Jackson laboratory) or their WT littermates (Fig. 1b). Furthermore, similar observations were made when mice were pre-conditioned with busulfan and cyclophosphamide (BU/CY) chemotherapy (Fig. 1c). *Caspase-11*-deficient recipients had markedly fewer pathological injuries (in liver, lung, small intestine, and colon) (Fig. 1d), and displayed significantly lower GVHD pathological scores compared with WT controls (Fig. 1e and Supplementary Fig. 1a). However, pre-transplant radiation resulted in similar weight loss and tissue injury in *Caspase-11*-deficient recipients and their WT controls (Supplementary Fig. 1b, c). We next examined whether caspase-11 in donor cells contributes to the pathogenesis of GVHD. Purified T cells and BM from *Caspase-11*-deficient mice or their WT littermates (on a B6 background, H2b) were transplanted into lethally irradiated recipient mice on a BALB/c background (H2d). Deletion of *Caspase-11* in donor cells resulted in a delayed mortality and slightly improved GVHD pathological scores in WT recipients (Fig. 1f and Supplementary Fig. 1d, e). We established that caspase-11 expressed in the non-hematopoietic compartment plays the dominant role in promoting GVHD lethality in allo-HSCT, while caspase-11 expressed in the hematopoietic compartment plays a minor role (Supplementary Fig. 1f). *Caspase-11* deficiency in both recipients and donor BM cells further decreased allogeneic T cell-mediated lethality (Supplementary Fig. 1f).

### Caspase-11 enhances donor T cell expansion

**Fig. 2:**
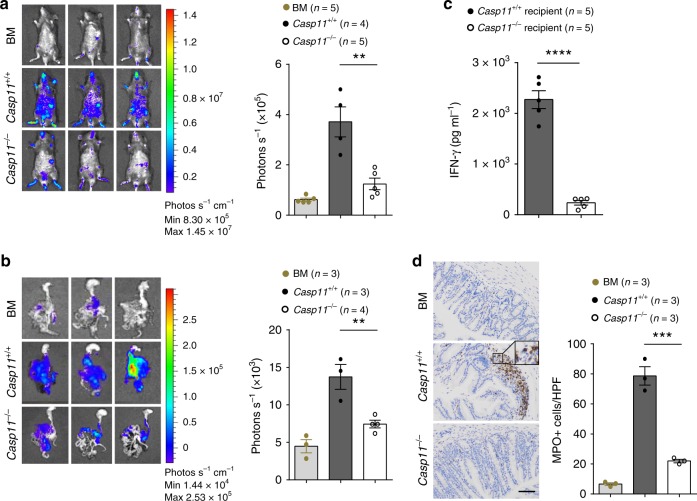
Caspase-11 enhances donor T cell expansion in allogeneic hematopoietic stem cell transplantation (allo-HSCT). **a** Fourteen days post transplant, *Casp11*^+/+^ and *Casp11*^−/−^ recipients receiving BALB/c bone marrow and T cells were subjected to intraperitoneal luciferin injection and whole-body bioluminescent imaging (BLI) are depicted. Region of interest gating and signal normalization with Living Image software. Average representative whole-body images, and average bioluminescence intensities ± SEM were depicted. (**P = 0.0040; an unpaired Student’s *t* test (two-sided) was used). **b** Fourteen days post transplant, *Casp11*^+/+^ and *Casp11*^−/−^ recipients receiving BALB/c bone marrow and T cells were subjected to intraperitoneal luciferin injection and whole-body BLI followed by a second luciferin injection, euthanization, organ removal, and BLI of graft-versus-host disease (GVHD) target organs of intestines. Bioluminescence was quantified using whole organ region of interest gating and signal normalization with Living Image software. Average representative ex vivo organ images, and average bioluminescence intensities ± SEM were depicted. (**P = 0.0089; an unpaired Student’s *t* test (two-sided) was used). **c** Interferon-γ (IFN-γ) in the serum of mice receiving BM and T cells (groups described in **a**, BALB/C (donor), C57BL/6 (recipient) model, day 5 after allo-HSCT). Data (mean ± SEM) are pooled from two independent experiments (****P < 0.0001, an unpaired Student’s *t* test (two-sided) was used). **d** Representative colon tissue section of *Casp11*^*+/+*^ recipient mice versus *Casp11*^−*/*−^ recipient mice (BALB/c → C57BL/6 combination) receiving BALB/c bone marrow and T cells is shown. Staining in brown is for myeloperoxidase as indicated for the respective tissues. The frequency of myeloperoxidase positive (MPO+) cells per high power field (HPF) was significantly lower in the caspase-11-deficient recipients than those of in wild-type (WT) recipients. Data are presented as mean ± SEM (***P = 0.0008, an unpaired Student’s *t* test (two-sided) was used). Scale bar: 100 μm

The tissue injury associated with GVHD is caused by the activation of allogeneic donor T cells. To test whether caspase-11 enhances allogeneic donor T cell expansion following HSCT, we used BALB/c donor T cells that express luciferase linked to the β-actin promoter. Two weeks after allo-HSCT, *Caspase-11*-deficient or WT recipients were subjected to bioluminescence (bioluminescent imaging (BLI)) to measure donor T cell expansion. Quantification of whole-body or intestinal BLI intensity indicated that *Caspase-11*-deficient recipients had significantly reduced T cell expansion, as compared to that of WT recipients (Fig. 2a, b). Activation of allogeneic T cells in GVHD is associated with increased production of interferon-γ (IFN-γ) and other proinflammatory cytokines, such as IL-17. Neutrophil recruitment into the tissues promotes allogeneic donor T cell expansion and exacerbates GVHD-related tissue injury and mortality^1^. In line with these findings, serum levels of IFN-γ and neutrophil infiltration in the colon of *Caspase-11*-deficient recipients were significantly lower than those in WT recipients (Fig. 2c, d). *Caspase-11* deficiency in recipient mice was also associated with significantly lower serum levels of IL-17 and IL-6 (Supplementary Fig. 2a–c) following allo-HSCT. Deletion of *Caspase-11* significantly inhibited CD4 T cell T-helper type 1 (Th1) and Th17 polarization in the intestine (Supplementary Fig 2d, e). Taken together, our data demonstrate that Caspase-11 promotes neutrophil infiltration, intestinal inflammation, tissue damage, donor T cell expansion and mortality in allo-HSCT.

### LPS–caspase-11 interaction enhances GVHD

**Fig. 3:**
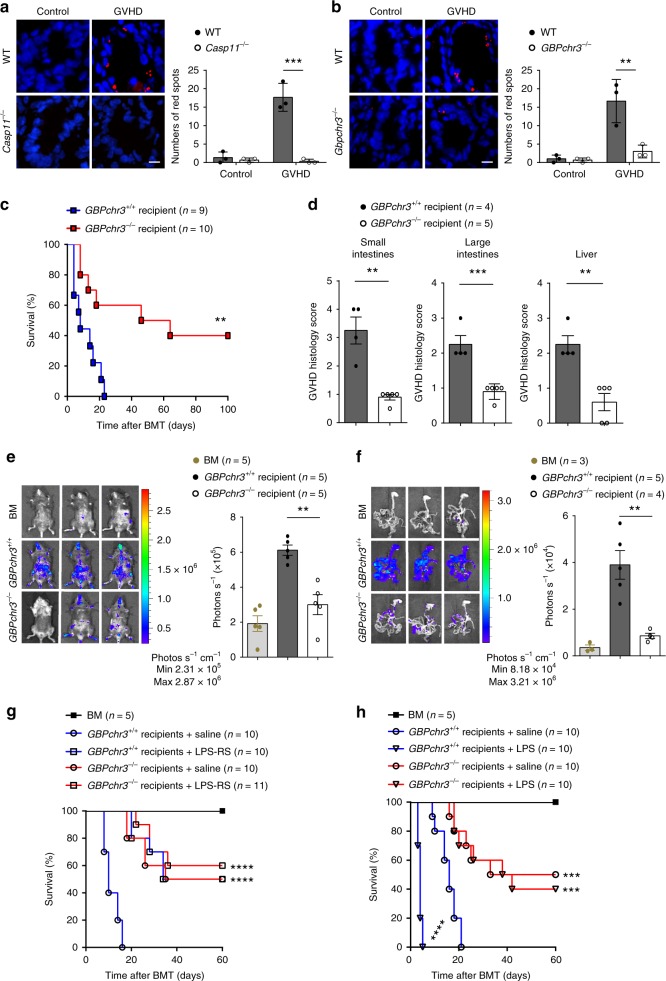
Lipopolysaccharide (LPS)–caspase-11 interaction enhances graft-versus-host disease (GVHD) in allogeneic hematopoietic stem cell transplantation (allo-HSCT). **a** The physical interactions between caspase-11 and LPS were visualized as the red spots by proximity-ligation assay (PLA) in the intestinal tissues in *Casp11*^+/+^ and *Casp11*^−/−^ recipient mice receiving BALB/c bone marrow and T cells. Scale bar: 10 μm. Data are presented as mean ± SEM (***P < 0.001, an unpaired Student’s *t* test (two-sided) was used). **b** The physical interactions between caspase-11 and LPS were visualized as the red spots by PLA in the intestinal tissues in *Gbpchr3*^*+/+*^ and *Gbpchr3*^−*/*−^ recipient mice receiving BALB/c bone marrow and T cells. Scale bar: 10 μm. Data are presented as mean ± SEM (**P < 0.01, an unpaired Student’s *t* test (two-sided) was used). **c** Survival of C57BL/6 mice is shown (*Gbpchr3*^*+/+*^ versus *Gbpchr3*^−*/*−^, **P = 0.0045; differences in animal survival were analyzed by log-rank test). **d** Histopathological GVHD severity of C57BL/6 mice after receiving BALB/c bone marrow and T cells (BALB/c → C57BL/6) is shown (*Gbpchr3*^*+/+*^ versus *Gbpchr3*^−*/*−^). Data are presented as mean (**P < 0.01; ***P < 0.001; an unpaired Student’s *t* test (two-sided) was used). **e** Fourteen days post transplant, *Gbpchr3*^*+/+*^ and *Gbpchr3*^−*/*−^ recipients were subjected to intraperitoneal luciferin injection and whole-body bioluminescent imaging (BLI) are depicted. Average representative whole-body images and bioluminescence intensities ± SEM are depicted (**P = 0.0013; an unpaired Student’s *t* test (two-sided) was used). **f** Fourteen days post transplant, *Gbpchr3*^*+/+*^ and *Gbpchr3*^−*/*−^ recipients were subjected to intraperitoneal luciferin injection, whole-body BLI, and BLI of intestines. Average representative ex vivo organ images and average bioluminescence intensities ± SEM are depicted (**P = 0.0034; an unpaired Student’s *t* test (two-sided) was used). **g** Percentage survival of *Gbpchr3*^*+/+*^ and *Gbpchr3*^−*/*−^ recipient mice receiving BALB/c bone marrow and T cells with treatment of lipopolysaccharide from the photosynthetic bacterium *Rhodobacter sphaeroides* (LPS-RS) or saline is shown. Differences in animal survival were analyzed by log-rank test. (*Gbpchr3*^+/+^ + saline versus *Gbpch3*^−/−^ + saline, ****P < 0.0001; *Gbpchr3*^*+/+*^ + saline versus *Gbpchr3*^+/+^ + LPS-RS, ****P < 0.0001; *Gbpchr3*^+/+^ + saline versus *Gbpchr3*^−/−^ + LPS-RS, ****P < 0.0001). **h** Percentage survival of *Gbpchr3*^*+/+*^ and *Gbpchr3*^−*/*−^ recipient mice with multiple injections of LPS or saline is shown. Differences in animal survival were analyzed by log-rank test. (*Gbpchr3*^*+/+*^ + saline versus *Gbpchr3*^−*/*−^ + saline, ***P = 0.0004.; *Gbpchr3*^*+/+*^ + saline versus *Gbpchr3*^*+/+*^ + LPS, ****P < 0.0001; *Gbpchr3*^*+/+*^ + saline versus *Gbpchr3*^−***/***−^ + LPS, ***P = 0.0002)

Caspase-11 is a cytosolic LPS receptor that is constitutively expressed in intestinal tissues^24^. To assess the LPS–caspase-11 interaction in allo-HSCT, we performed the proximity-ligation assay (PLA) that visualizes the physical interaction between two molecules^22^. Allo-HSCT markedly increased the LPS–caspase-11 interaction in the intestine of WT mice (Fig. 3a). As expected, the interaction was lost with genetic deletion of *Caspase-11* (Fig. 3a). LPS–caspase-11 complexes predominantly co-localized with an epithelial marker (Supplementary Fig. 3a), further supporting the notion that caspase-11 expressed in the non-hematopoietic compartment plays the dominant role in promoting GVHD following allo-HSCT. Recognition of LPS by caspase-11 requires guanylate-binding proteins (GBPs)^13,25^. In line with these findings, LPS–caspase-11 interaction in the intestinal epithelium was not observed when GBPchr3 KO (knockout) mice^13^, which lack GBP1, 2, 3, 5, and 7, were used as HSCT recipients (Fig. 3b). We also observed that deletion of GBPs in recipients significantly improved survival (Fig. 3c), and markedly reduced serum levels of IFN-γ as well as neutrophil infiltration into the colon (Supplementary Fig. 3b, c). Accordingly, GBPchr3 KO recipients displayed significantly improved GVHD pathological scores as compared to WT recipients (Fig. 3d and Supplementary Fig. 3d). GBPchr3 KO recipients also exhibited reduced T cell expansion to a level similar to that seen in *Caspase-11*-deficient recipients (Fig. 3e, f).

To further confirm that the interaction of LPS with caspase-11 promotes GVHD, mice were treated with LPS from the photosynthetic bacterium *Rhodobacter sphaeroides* (LPS-RS), the penta-acylated LPS that competitively inhibits the LPS–caspase-11 interaction^22^ (Supplementary Fig. 3a). Treatment of LPS-RS significantly promoted survival in WT but not GBPchr3 KO recipient mice following allo-HSCT (Fig. 3g). Conversely, intraperitoneal (i.p.) administration of LPS after allo-HSCT significantly promoted GVHD lethality in WT but not in *Caspase-11*-deficient or GBPchr3 KO recipient mice (Fig. 3h). Oral administration of ciprofloxacin significantly decreased the circulating LPS levels (Supplementary Fig. 4a, b). Accordingly, ciprofloxacin treatment significantly improved survival in WT but not in *Caspase-11*-deficient recipient mice (Supplementary Fig. 4c). Taken together, these data indicate that LPS–caspase-11 interaction enhances GVHD in allo-HSCT.

### Caspase-11 enhances GVHD through GSDMD

**Fig. 4:**
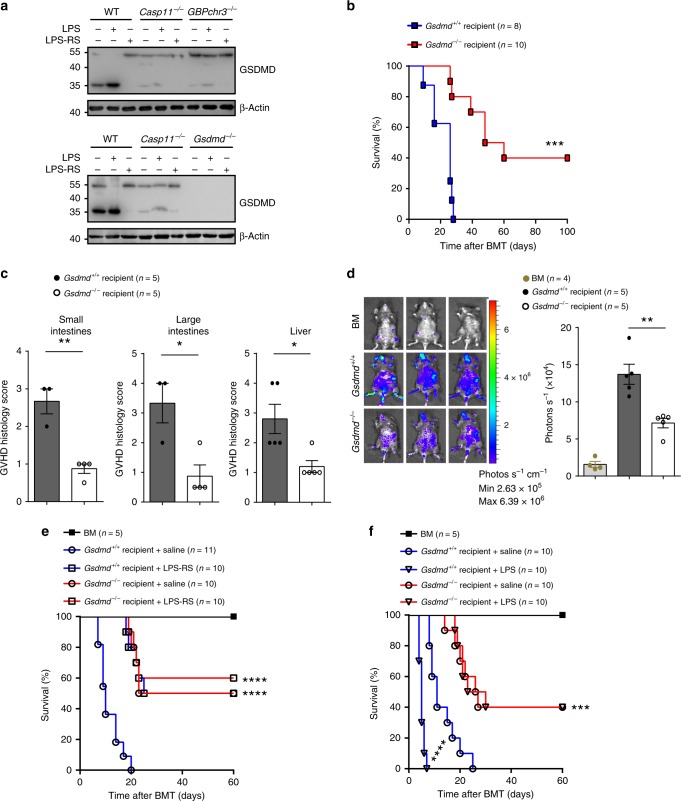
Caspase-11 enhances graft-versus-host disease (GVHD through gasdermin D (GSDMD) in allogeneic hematopoietic stem cell transplantation (allo-HSCT). **a** Immunoblot for GSDMD and β-actin in the intestine from wild-type (WT) (C57BL/6), *Gbpchr3*^−*/*−^ or Gsdmd^−/−^ recipients mice with treatment of lipopolysaccharide (LPS) or lipopolysaccharide from the photosynthetic bacterium *Rhodobacter sphaeroides* (LPS-RS). **b** Survival of Gsdmd^+/+^ versus Gsdmd^−/−^ recipient mice (BALB/c → C57BL/6 combination) receiving BALB/c bone marrow and T cells is shown. The experiment was performed twice and the results were pooled. Gsdmd^+/+^ versus Gsdmd^−/−^, ***P *=* 0.0002; differences in animal survival were analyzed by log-rank test. **c** Histological analysis of the small intestines, large intestines, and liver from Gsdmd^+/+^ and Gsdmd^−/−^ recipient mice receiving BALB/c bone marrow and T cells for GVHD severity on day 14. Data are presented as mean ± SEM. **P < 0.01; *P < 0.05. An unpaired Student’s *t* test (two-sided) was used. **d** Fourteen days post transplant, Gsdmd^+/+^ and Gsdmd^−/−^ recipients receiving BALB/c bone marrow and T cells were subjected to intraperitoneal luciferin injection and whole-body bioluminescent imaging (BLI) are depicted. Average representative whole-body images and average bioluminescence intensities ± SEM were depicted. Data are presented as mean ± SEM. An unpaired Student’s *t* test (two-sided) was used. **P < 0.01(compared with vehicle-treated controls). **e** Percentage survival of *Gsdmd*^*+/+*^ and *Gsdmd*^−*/*−^ recipient mice receiving BALB/c bone marrow and T cells (BALB/c → C57BL/6 combination) with treatment of LPS-RS or saline is shown. Differences in animal survival were analyzed by log-rank test. (*Gsdmd*^*+/+*^ + saline versus *Gsdmd*^−*/*−^ + saline, ****P < 0.0001; *Gsdmd*^*+/+*^ + saline versus *Gsdmd*^*+/+*^ + LPS-RS, ****P < 0.0001; *Gsdmd*^*+/+*^ + saline versus *Gsdmd* ^−*/*−^ + LPS-RS, ****P < 0.0001). **f** Percentage survival of *Gsdmd*^*+/+*^ and *Gsdmd*^−*/*−^ recipient mice receiving BALB/c bone marrow and T cells (BALB/c → C57BL/6 combination) with multiple injections of LPS or saline is shown. Differences in animal survival were analyzed by log-rank test. (*Gsdmd*^*+/+*^ + saline versus *Gsdmd*^−*/*−^ + saline, ***P = 0.0005; *Gsdmd*^*+/+*^ + saline versus *Gsdmd*^*+/+*^ + LPS, ****P < 0.0001; *Gsdmd*^*+/+*^ + saline versus *Gsdmd*^−*/*−^ + LPS, ***P = 0.0004)

GSDMD is a caspase-11 substrate that directly triggers pyroptosis^16,17^. Allo-HSCT leads to the cleavage of GSDMD in the intestine in WT recipients. Deletion of *Caspase-11* or GBPs in recipient mice markedly decreased GSDMD cleavage in allo-HSCT (Fig. 4a). Treatment of LPS-RS significantly inhibited and LPS administration enhanced GSDMD cleavage in the intestine after allo-HSCT (Fig. 4a). Next, we determined whether caspase-11 enhances GVHD through GSDMD in the MHC-mismatched allo-HSCT. Deletion of *Gsdmd* significantly improved survival (Fig. 4b), and markedly reduced serum IFN-γ levels (Supplementary Fig. 5a) and intestinal neutrophil infiltration (Supplementary Fig. 5b). *Gsdmd* deficiency significantly improved GVHD pathological scores in a manner similar to *Caspase-11* deficiency (Fig. 4c and Supplementary Fig. 5c) and was associated with reduced T cell expansion as using luciferase-expressing T cells as described above (Fig. 4d). Treatment of LPS-RS significantly promoted survival in WT but not in *Gsdmd* KO recipient mice during allo-HSCT (Fig. 4e). Further, LPS treatment significantly promoted lethality in WT but not in *Gsdmd* KO recipient mice during allo-HSCT (Fig. 4f). These findings show that caspase-11 promotes GVHD through GSDMD in allo-HSCT.

### Caspase-11 and GSDMD mediates IL-1α release in GVHD

**Fig. 5:**
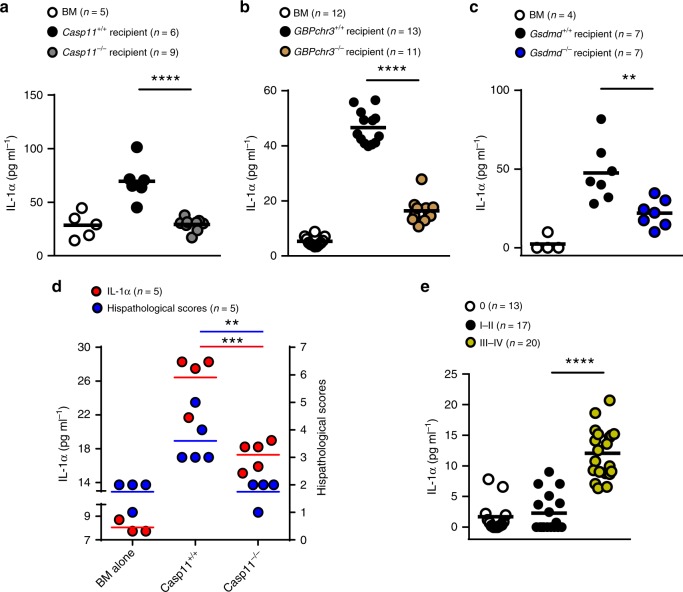
Caspase-11 and gasdermin D (GSDMD) mediate interleukin-1α (IL-1α) release in graft-versus-host disease (GVHD). **a**–**c** Total body irradiation (1100 cGy) was dosed to C57BL/6 mice, which were then transplanted with 5 × 10^6^ wild-type (WT) BALB/c T cell-depleted bone marrow (TCD-BM) cells alone or in addition to 5 × 10^6^ total T cells on day 0. Five days post transplant, serum was subjected to cytokine analysis to quantify serum cytokine concentrations of IL-1α in *Casp11*^−/−^ (**a**) *Gbpchr3*^−*/*−^ (**b**) and *Gsdmd*^−/−^ (**c**) recipients. An unpaired Student’s *t* test (two-sided) was used. **P < 0.01; ****P < 0.0001 (compared with vehicle-treated controls). **d** The serum IL-1α levels of *Casp11*^*+/+*^ and *Casp11*^−*/*−^ recipients that had undergone allogeneic hematopoietic stem cell transplantation (allo-HSCT) was correlated with GVHD severity. An unpaired Student’s *t* test (two-sided) was used. **P < 0.01; ***P < 0.001. **e** The levels of IL-1α in the serum was correlated with the GVHD severity (GVHD grade 0, *n* = 13; GVHD grade I–II, *n* *=* 17; GVHD grade III–IV, *n* *=* 20) in patients after allo-HSCT. ****P < 0.0001. An unpaired Student’s *t* test (two-sided) was used. The patients’ characteristics are detailed in Supplementary Table 1

Caspase-11 activation and subsequent GSDMD cleavage lead to the release of IL-1α in endotoxemia and bacterial sepsis^10,16,22^. We next determined whether caspase-11 and GSDMD mediates IL-1α release in GVHD. Serum levels of IL-1α were significantly increased in allo-HSCT (Fig. 5a–c). Deletion of *Caspase-11* or *Gsdmd* in recipients markedly reduced IL-1α release, indicating that caspase-11- and GSDMD-expressing non-hematopoietic cells (e.g., epithelial cells) are the major source of IL-1α after allo-HSCT (Fig. 5a–c). Serum levels of IL-1α were correlated with GVHD pathological scores in mouse models (Fig. 5d). Importantly, we prospectively analyzed the serum levels of 50 patients undergoing allo-HSCT (Supplementary Table 1) and found that the serum levels of IL-1α in patients with severe GVHD were significantly higher than those in patients with no or mild GVHD (Fig. 5e). Together, these findings indicate that caspase-11 and GSDMD mediates IL-1α release in GVHD.

### Neutralizing IL-1α attenuates GVHD

**Fig. 6:**
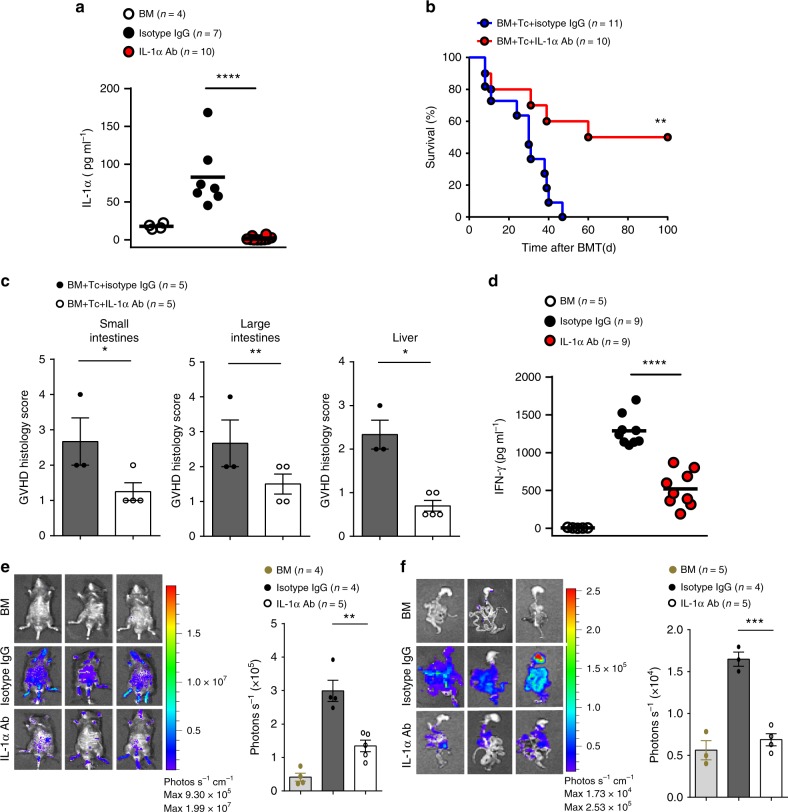
Neutralizing interleukin-1α (IL-1α) attenuates graft-versus-host disease (GVHD). **a** Levels of IL-1α in the serum are shown from mice bone marrow (BM) receiving BALB/c BM or injected with 4 µg per mouse anti-IL-1α monoclonal antibody (Ab) or isotype control Ab per mouse on day −1 and day +1 of allogeneic hematopoietic stem cell transplantation (allo-HSCT). Scatter plot represent mean, *n* = 4 (BM), *n* = 10 (IL-1α Ab) and *n* = 7 (isotype control) with results pooled from two independent experiments. ****P < 0.0001, unpaired Student’s *t* test (two-sided). **b** Survival of C57BL/6 recipients (BALB/c → C57BL/6 combination) is shown for a group that received BM/Tc (BM/T cell) with anti-IL-1α monoclonal Ab or isotype control Ab. BM/Tc + isotype control Ab (*n* = 11) versus BM/Tc + anti-IL-1α monoclonal Ab (*n* = 10), **P *=* 0.0065. Differences in animal survival were analyzed by log-rank test. **c** Histological analysis of the small intestines, large intestines, and liver from anti-IL-1α monoclonal Ab and isotype control Ab mice for GVHD severity on day 14. Data are presented as mean ± SEM. An unpaired Student’s *t* test (two-sided) was used. *P < 0.05; **P < 0.01. **d** Levels of interferon-γ (IFN-γ) in the serum of the BM/Tc + isotype control Ab (*n* *=* 9) and BM/Tc + anti-IL-1α monoclonal Ab recipient mice (*n* *=* 9) on the day 5 after allo-HSCT. ****P < 0.0001. An unpaired Student’s *t* test (two-sided) was used. **e** Fourteen days post transplant, BM/Tc + isotype control Ab and BM/Tc + anti-IL-1α monoclonal Ab recipients were subjected to intraperitoneal luciferin injection and whole-body bioluminescent imaging (BLI) are depicted. Average representative whole-body images, and average bioluminescence intensities ± SEM were depicted. An unpaired Student’s *t* test (two-sided) was used. **P < 0.01 (compared with vehicle-treated controls). **f** Fourteen days post transplant, BM/Tc + isotype control Ab and BM/Tc + anti-IL-1α monoclonal Ab recipients were subjected to intraperitoneal luciferin injection, whole-body BLI and BLI of intestines. Average representative ex vivo organ images, and average bioluminescence intensities ± SEM were depicted. An unpaired Student’s *t* test (two-sided) was used. ***P = 0.0003 (compared with vehicle-treated controls)

Next, we tested whether caspase-11 signaling enhances GVHD in allo-HSCT through releasing IL-1α. Neutralizing IL-1α using monoclonal antibodies, which almost completely depleted IL-1α in serum (Fig. 6a), led to a marked improvement of GVHD mortality and a significant reduction of GVHD pathological scores (Fig. 6b, c). Meanwhile, administration of IL-1α-neutralizing antibodies significantly decreased serum IFN-γ levels (Fig. 6d), and reduced donor T cell expansion in the whole body and small intestine as compared to their WT littermates (Fig. 6e, f). Importantly, neutralizing IL-1α significantly improved survival in allo-HSCT in WT mice but not in *Caspase-11*-deficient recipients (Supplementary Fig. 6a). Caspase-11- and GSDMD-induced pyroptosis leads to potassium efflux, resulting in the activation of NLRP3 inflammasome^16^. This event mediates the maturation of IL-1β, which has similar biological functions as IL-1α. In agreement with a previous study^26^, *Nlrp3* deficiency in recipients delayed but did not reduce mortality when mice were injected with 10^6^ allogeneic donor T cells, whereas *Caspase-11*-deficiency significantly reduced mortality in the same experimental setting (Supplementary Fig. 6b). Caspase-11 and GSDMD were also critical for IL-18 release in allo-HSCT (Supplementary Fig. 6c, d). However, genetic deletion of *Il-18* failed to affect the survival (Supplementary Fig. 6e). Together, these observations suggest that caspase-11 signaling enhances GVHD, at least in part, through IL-1α.

We next investigated the mechanisms by which IL-1α promotes donor T cell expansion and GVHD severity. Administration of IL-1α-neutralizing antibodies markedly reduced intestinal neutrophil infiltration in allo-HSCT (Supplementary Fig. 7a). In agreement with a previous study^1^, depletion of neutrophils using anti-Ly-6G antibodies significantly improved survival and markedly inhibited donor T cell expansion in allo-HSCT (Supplementary Fig. 7b, c). These findings suggest that IL-1α promotes neutrophil infiltration in allo-HSCT, which is critical for donor T cell expansion and GVHD severity.

### *Caspase-11* deficiency preserves GVL activity

**Fig. 7:**
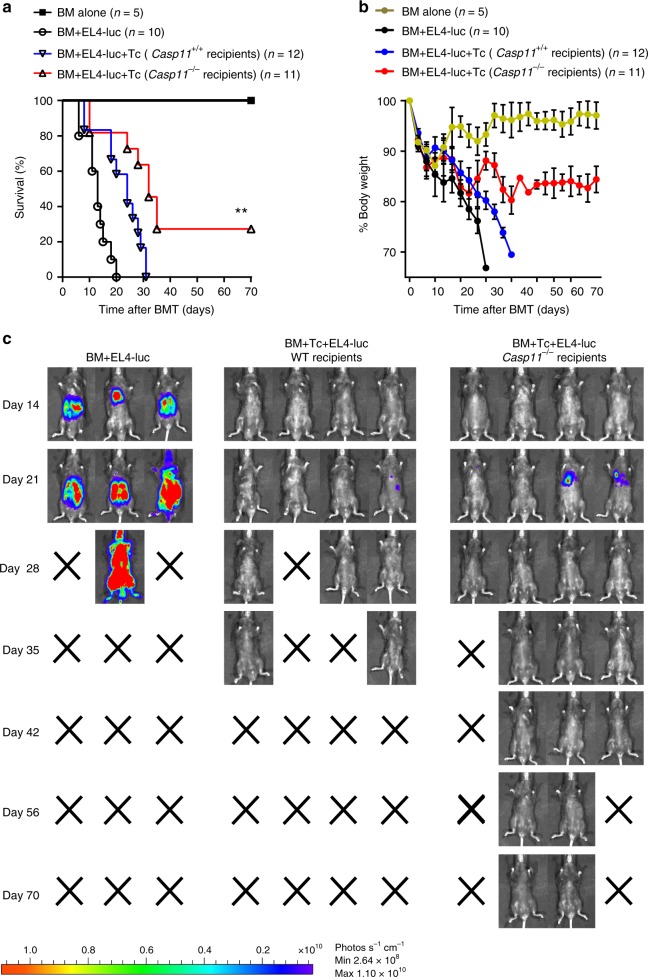
*Caspase-11* deficiency preserves graft-versus-leukemia (GVL) activity. **a** Survival of *Casp11*^*+/+*^ (*n* = 12) and *Casp11*^***−****/*−^ (*n* = 11) recipients had received lethally irradiation (1100 cGy) and transplanted with 5 × 10^6^ BALB/c T cell-depleted bone marrow (TCD-BM) cells alone or in addition to 3 × 10^6^ total T cells from BALB/c mice with 3 × 10^5^ EL4 luciferase-transduced lymphoma cells at the time BM transplant (BMT) was shown. Differences in animal survival were analyzed by log-rank test. **P = 0.0039 (*Casp11*^*−/*−^ compared with vehicle-treated controls). **b** *Casp11*^*+/+*^ (*n* = 12) and *Casp11*^−*/*−^ (*n* = 11) recipients were monitored throughout the experimental period for weight change by every 3 to 5 days. **c** Tumor expansion in *Casp11*^*+/+*^ and *Casp11*^−*/*−^ recipients was shown by luciferin intraperitoneal injection and whole-body bioluminescent imaging (BLI) was taken every 14 days

GVL activity is essential for preventing tumor relapse, and is the key to the success of allo-HSCT^27^. Thus, we next determined whether *Caspase-11* deficiency in recipients affects GVL maintenance by using allo-HSCT model supplemented with EL4 murine T lymphoma cells transduced with luciferase/neo plasmid (EL4-luc) for BLI tumor tracking. Six weeks after MHC-mismatched allo-HSCT, GVHD versus tumor mortality was distinguished by the BLI signal (indicative of tumor load) and weight loss (indicative of GVHD onset). As expected, all recipients of EL4-luc and BM succumbed to tumor within 4 weeks (Fig. 7a). WT recipients of allogeneic T cells all succumbed to GVHD by day 30 after allo-HSCT in parallel with notable weight loss (Fig. 7b). These mice lacked a tumor BLI signal and visible tumor (Fig. 7c). Deletion of *Caspase-11* in recipients significantly improved both weight maintenance and survival (Fig. 7a–c). Remarkably, none of the *Caspase-11*-deficient mice succumbed to tumor (Fig. 7a and Supplementary Fig. 8). These findings demonstrate that *Caspase-11* deficiency does not compromise the GVL effect in allo-HSCT.

## Discussion

Taken together, this study establishes that blocking caspase-11 signaling reduces GVHD while preserving GVL activity. In this scenario, circulating LPS or Gram-negative bacteria activate caspase-11 through GBPs, leading to GSDMD-dependent IL-1α release. Targeting this pathway by genetic or pharmacological approaches reduced intestinal neutrophil infiltration, tissue damage, and donor T cell expansion in allo-HSCT. Together with a recent finding that gut microbiota-induced neutrophil recruitment to the intestinal tissue promotes donor T cell activation and contributes to the pathogenesis of GVHD^1^, our data shed mechanistic insights into how gut microbiota promotes GVHD in allo-HSCT. The important contribution of gut microbiota to GVHD was first discovered in the 1970s. It then became a common practice to perform intestinal decontamination using orally administered antibiotics in patients undergoing allo-HSCT^27^. Initial reports were promising, but subsequent studies could not confirm a benefit^27^. Broad-spectrum antibiotic use even promotes GVHD by causing dysbiosis^28^. Furthermore, gut microbiota-derived metabolites, such as butyrate, improve intraepithelial cell junctional integrity, decreases apoptosis, and mitigates GVHD^29^. Therefore, pharmacologically targeting gut microbiota-induced inflammatory responses, such as the caspase-11 signaling, might be a more effective option for patients undergoing allo-HSCT than eliminating bacterial colonies in the GI tract through antibiotics.

Type 1 IFN signaling is important for the activation of caspase-11^11,13,15^. Priming with poly I:C, a double-stranded RNA mimetic that induces robust type 1 IFN production in a manner similar to that of double-stranded RNA virus, could significantly promote LPS-induced caspase-11 activation and pyroptosis in lung endothelial cells^21^. Further, administration of poly I:C markedly increases caspase-11-dependent lethality in endotoxemia^19,20^. Recent advances reveal a progressive expansion of viral infections over time following allo-HSCT^30^. In particular, the picobirnaviruses, which are double-stranded RNA virus, are strongly associated with severe enteric GVHD^30^. These findings raise an intriguing possibility that allo-HSCT-associated viral infections might enhance GVHD through the caspase-11-GSDMD signaling. It is noteworthy that caspase-1 also mediates GSDMD cleavage. Both gut microbiota and viral infections are able to activate caspase-1 through canonical inflammasomes. Thus, the roles of caspase-1 in the pathogenesis of GVHD in allo-HSCT merit further investigations.

GVHD remains a major obstacle for the wider usage of allo-HSCT^1,2^. To date, most therapeutic or prophylactic strategies to prevent severe GVDH include T cell depletion, pharmacological inhibition of T cell activation and proliferation, and intervene with the T cell-dependent effector phase^27^. These approaches inevitably impair the global host immunity, and thus compromise the GVL activity mediated by donor T cells, which is the major determinant of the overall outcome after allo-HSCT^27^. In the current study, we show that the absence of caspase-11 signaling reduces GVHD while preserving GVL activity. Therefore, our data have major implications for allo-HSCT, as pharmacological inhibition of caspase-11 signaling might exclusively reduce the unwanted graft-versus-host reactions while sparing the beneficial GVL activity in allo-HSCT.

## Methods

### Human subjects

Serum samples were collected at 30 to 80 days after GVHD diagnosis, in accordance with research ethics board approval from Xiangya Hospital of Central South University (CSU). All patients provided informed consent to data collection for research. Patient characteristics are given in Supplementary Table 1. GVHD grading was performed on the basis of Glucksberg grade standard.

### Mice

C57BL/6 (H-2Kb, Thy-1.2) and BALB/c (H-2Kd, Thy-1.2) mice were purchased from Hunan SJA Laboratory Animal Co. (Changsha, China). *Caspase11*^−/−^, *Il-18*^−*/*−^, and L2G85.BALB/c mice were purchased from Jackson laboratory. The *Caspase11*^−*/*−^ mice^22^ were donated by Timothy R. Billiar (Department of Surgery, University of Pittsburgh Medical Center, Pittsburgh, PA). The *GBPchr3*^−*/*−^ mice^31^ were donated by Petr Broz (Focal Area Infection Biology, Biozentrum, University of Basel). The *Gsdmd*^−*/*−^ mice^32^ were donated by Jiahuai Han (State Key Laboratory of Cellular Stress Biology Innovation Center for Cell Signaling Network School of Life Sciences, Xiamen University). The *Nlrp3*^−*/*−^ mice^33^ were donated by Rongbin Zhou (CAS Key Laboratory of Innate Immunity and Chronic Disease, School of Life Sciences, University of Science and Technology of China). All mice except the L2G85.BALB/c mice used in this study are on C57BL/6 background and were housed in specific pathogen-free conditions in the Department of laboratory animals of CSU in China. Mice were used between 6 and 12 weeks of age, and only sex-matched donor–recipient pairs were used. All work was approved and performed according to the Guidelines for Animal Experiments by the Institutional Animal Care and Use Committee of Central South University.

### BMT model

BM transplant recipients were given 5 × 10^6^ BM cells (Biotin antibody CD90.2, eBioscience; MACS negative sorting, STEMCELL, Canada) after lethal irradiation with 11 Gy (split doses of 2 × 5.5 Gy apart to 4 h) for the BALB/c → C57BL/6 combination and 8 Gy (split doses of 2 × 4.0 Gy apart to 4 h) for the C57BL/6 → BALB/c combinations at CSU, China. T cell doses (Biotin antibodies CD11b/TER119/B220/CD49b/CD24/CD19b, eBioscience; MACS negative enrichment, STEMCELL, Canada) varied depending on the transplant model C57Bl/6 ≥ BALB/c (8 × 10^5^) and BALB/c ≥ C57Bl/6 (2–5 × 10^6^).

### Histopathology scoring of acute GVHD

Slides of small bowel and large bowel samples collected on day 14 were stained with hematoxylin/eosin and scored by experienced pathologists blinded to the treatment groups. Our GVHD grading system was as follows: liver—grade 0.5, focal portal lymphoid infiltrate; grade 1, widespread portal lymphoid infiltrate; grade 2, focal bile duct invasion or cellular injury; grade 3, multiple foci of bile duct injury and regeneration; grade 4, widespread injury and destruction of bile ducts. Small and large intestines—grade 0.5, occasional or rare necrotic cells in glands or crypts; grade 1, multiple foci of necrotic cells in glands or crypts; grade 2, necrosis involving several crypts or glands with focal abscess formation in crypts; grade 3, widespread crypt abscesses with focal glandular destruction; grade 4, loss of mucosa with granulation tissue response.

### T cell lymphoma model

To investigate GVL activity of transferred donor T cells, we used luciferase transgenic EL4 (EL4-luc) T cell leukemia that had been previously demonstrated to migrate primarily to the BM, with secondary infiltration of the spleen and other lymphoid organs when injected intravenously at the time of BMT. BALB/c → B6 recipients received 5 × 10^3^ luciferase/neo plasmid-transduced EL4 T cell lymphoma cells (EL4-luc) at the time of BMT. Tumor mortality and GVHD mortality were distinguished by BLI for tumor load and weight loss indicative of GVHD. BLI data were analyzed and quantified using the Living Image Software (Xenogen).

### Chemotherapy treatment

Mice received BU (10 mg kg^−1^ day^−1^ from days –7 to –4; Busilvex, Pierre Fabre Pharma, Germany) and CY (100 mg kg^−1^ day^−1^ from days −3 to −2; Endoxan, Baxter Healthcare, USA) i.p. (BU/CY). Day of stem cell injection was assigned as day 0 and the days before stem cell injection are numbered backwards.

### Drug treatment

The mice were injected i.p. with 4 µg per mouse a monoclonal anti-mouse IL-1α antibody (Ab) (BioLegend, Germany) or isotype control Ab (rat IgG2a, BioLegend, Germany) in 100 µl of NS 24 h before TBI (total body irradiation) and 24 h after BMT.

### BLI imaging

For BLI studies, mice were injected i.p. with luciferin (10 µg g^−1^ body weight). Ten minutes later mice were imaged using an IVIS100 charge-coupled device (CCD) imaging system (Xenogen, Alameda, CA) for 5 min. Expansion was quantified in photons s^–1^ cm^–2^. Imaging data were analyzed and quantified with Living Image Software (Xenogen)

### Conventional histology and immunohistochemistry

Paraffin-embedded sections of 5-µm thickness were mounted on microscope slides. After fixing, the primary Ab (anti-MPO Abs, Abcam, USA) was applied. Sections were then incubated with a specific secondary biotinylated Ab. Streptavidin horseradish peroxidase and 3,3′-diaminobenzidine (Abcam, USA) were used according to the manufacturer’s instructions, and sections were counterstained with hematoxylin (Thermo Scientific, USA).

### Cytokine measurements

The levels of IL-1α and IFN-γ were analyzed from the serum with the eBioscience Inflammation Kit (eBioscience, USA) and used according to the manufacturer’s instructions.

### Isolation of lamia propria lymphocytes

Mice were sacrificed and intestines were flushed with PBS^Ca-/Mg-^ after dissection of fat and mesenteric tissue and excision of Peyer’s patches. Pieces (0.5 cm) of longitudinally opened and PBS^Ca-/Mg^-washed intestine were incubated with Hank's balanced salt solution (HBSS) containing 2 mM EDTA, 10 mM HEPES, 5% fetal bovine serum (FBS), and 1 mM dithiothreitol. After 15 min of incubation on a shaker at 37 °C, intraepithelial lymphocytes and epithelium were separated via 100-µm filters. Tissues were digested in the HBSS^Ca+/Mg+^ medium (Thermo Fisher Scientific) containing 10 mM HEPES, and 5% FBS at 37 °C in a 5% CO_2_ incubator for 0.5 h. Then, intestinal lamia propria lymphocytes (LPLs) were isolated using the gentleMACS Program m_intestine (gentleMACS Dissociator #130-093-235) and Intestinal LPL Isolation Kit (Lot: 130-097-410, Miltenyi, Germany).

### Flow cytometry

Staining was performed in the presence of purified Ab to CD16/32 at saturation to block nonspecific staining. For flow analysis, cells were lysed and fixed with 1× PhosphoFlow Lyse/Fix Buffer for 15 min at 37 °C, and then permeabilized with PhosFlow Perm Buffer III on ice for 1 h (all reagents were from eBioscience USA).

### Proximity-ligation assay

PLA Kit (Sigma, USA) was used to study the interaction between LPS and caspase-11 in mouse intestinal tissue. This unique method is able to visualize molecular interactions in situ. After fixation with 4% formaldehyde and permeabilization with 0.1% Triton for 10 min, cells were then incubated overnight with primary antibodies against LPS (mouse monoclonal 2D7/1) or caspase-11 (rat monoclonal 17D9). Briefly, after incubation with primary antibodies, cells were incubated with a combination of corresponding PLA probes, and secondary antibodies were conjugated to oligonucleotides (mouse PLUS and rat MINUS for LPS and caspase-11 interaction). Subsequently, ligase was added forming circular DNA strands when PLA probes were bound in close proximity, along with polymerase and oligonucleotides to allow rolling circle amplification. Fluorescent dye-labeled probes complementary in sequence to the rolling circle amplification product were hybridized to the rolling circle amplification product. Thus, each individual pair of proteins generated a spot that could be visualized using fluorescent microscopy. Images were taken using a Leica confocal laser scanning microscope and analyzed using the ImageJ software. In some experiments, cells were incubated with antibodies against mouse pan-cytokeratin (Clone: AE1/AE3, Lot: 2086277; Thermo, USA), the epithelial marker, after PLA procedure.

### Immunoblot

Proteins were extracted from intestinal tissues. Protein samples were separated by 12% sodium dodecyl sulfate-polyacrylamide gel electrophoresis and transferred onto PVDF membranes (Millipore). Antibodies against mouse GSDMD (Abcam, USA) was used at 1:1000. Blots were normalized to β-actin (CST, USA) expression.

### Statistical analyses

All data were analyzed using the GraphPad Prism software (version 6.01). Data were analyzed using Student’s *t* test for comparison between two groups. Survival data were analyzed using the log-rank test. A *p* value < 0.05 was considered statistically significant for all experiments. All values are presented as the mean ± SEM (error bars).

## Supplementary information

Supplementary Information

## Data Availability

The authors declare that all data supporting the findings of this study are available within the article and its Supplementary Information Files or from the corresponding author upon reasonable request.
