# Author Correction: Caspase-11 signaling enhances graft-versus-host disease

**DOI:** 10.1038/s41467-020-15161-8

**Published:** 2020-03-09

**Authors:** Yanyan Lu, Ran Meng, Xiangyu Wang, Yajing Xu, Yiting Tang, Jianfeng Wu, Qianqian Xue, Songlin Yu, Mingwu Duan, Dongyong Shan, Qingde Wang, Haichao Wang, Timothy R. Billiar, Xianzhong Xiao, Fangping Chen, Ben Lu

**Affiliations:** 1 Department of Hematology and Critical Care Medicine, The 3rd Xiangya Hospital, Central South University, Changsha, 410000 PR China; 2 Department of Hematology, Xiangya Hospital, Central South University, Changsha, 410008 PR China; 3 Department of Physiology, School of Basic Medical Science, Central South University, Changsha, 410000 Hunan PR China; 4 State Key Laboratory of Cellular Stress Biology Innovation Center for Cell Signaling Network School of Life Sciences, Xiamen University, Xiamen, 361005 Fujian PR China; 5 Department of Surgery, University of Pittsburgh Medical Center, Pittsburgh, PA 15213 USA; 6 The Feinstein Institute for Medical Research, Northwell Health, 350 Community Drive, Manhasset, NY 11030 USA; 7 Key Laboratory of Sepsis Translational Medicine of Hunan, Central South University, Changsha, 410000 Hunan PR China; 8 Department of Pathophysiology, School of Basic Medical Science, Central South University, Changsha, 410000 Hunan PR China

**Keywords:** Cancer immunotherapy, Bone marrow transplantation, Cell death and immune response, Inflammasome, Allotransplantation

Correction to: *Nature Communications* 10.1038/s41467-019-11895-2, published online 06 September 2019.

In the original version of this Article, the following author affiliations were inadvertently switched. Affiliation of Qingde Wang and Timothy R. Billiar should be: “Department of Surgery, University of Pittsburgh Medical Center, Pittsburgh, PA, 15213 USA”. Haichao Wang's affiliation should be: “The Feinstein Institute for Medical Research, Northwell Health, 350 Community Drive, Manhasset, NY 11030, USA”. The errors have been corrected in the HTML and PDF versions of this article.

