# Supplementary material for: Caspase-11 signaling enhances graft-versus-host disease
Source: Nat Commun. 2019 Sep 6;10:4044. doi: 10.1038/s41467-019-11895-2 (PMC6731232; doi:10.1038/s41467-019-11895-2)

# Cover Page

# Caspase-11 signaling enhances graft-versus-host disease

**Lu et al.**

Supplementary Figure 1

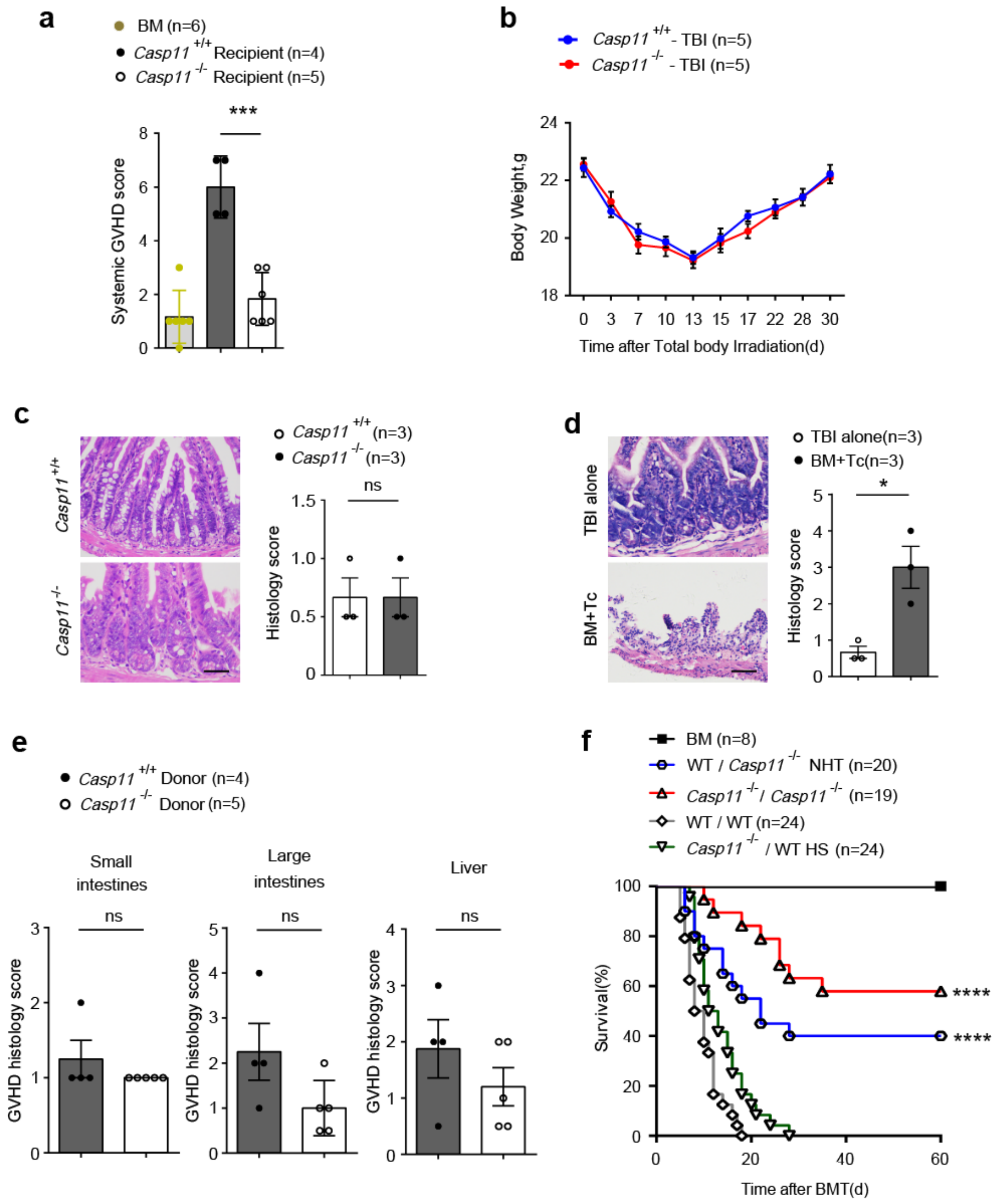


**Supplementary Figure 1: Caspase-11 enhances GVHD in allo-HSCT. a** Systemic GVHD scoring of the mice after transplanting with allogenic donor T cells for GVHD severity is depicted. Data are presented as mean ± SEM. ****P*=0.0003 unpaired Student's *t*-test (two-sided). **b** The body weight variation of *Casp11*$^{+/+}$ and *Casp11*$^{-/-}$ mice after total body radiation (TBI) and bone marrow transplantation is shown (n=5

in each group). **c** Photomicrographs depicting the morphology and histological analysis of the small intestines from *Casp11*$^{+/+}$ and *Casp11*$^{-/-}$ mice after total body radiation (TBI) and bone marrow transplantation are shown. Data are presented as mean ± SEM. *ns*, unpaired Student's *t*-test (two-sided). Scale bar: 100 μm. **d** Photomicrographs depicting the morphology and histological analysis of the small intestines from WT mice with TBI alone or transplantation with allogenic donor T cells. Data are presented as mean ± SEM. *$P$=0.0178, unpaired Student's *t*-test (two-sided). Scale bar: 100 μm. **e** Histological analysis of the small intestines, large intestines and liver from mice transplanted with *Casp11*$^{+/+}$ and *Casp11*$^{-/-}$ donor cells for GVHD severity on day 14. **f** Survival of bone marrow chimeras with *Casp11*$^{-/-}$ hematopoietic system (*Casp11*$^{-/-}$ /WT HS, the genotype in the left represents the hematopoietic system, the genotype in the right represents the non-hematopoietic compartment), or *Casp11*$^{-/-}$ non-hematopoietic tissue (WT / *Casp11*$^{-/-}$ NHT), or WT HSCT mice (WT / WT), or *Casp11*$^{-/-}$ mice reconstituted with *Casp11*$^{-/-}$ bone marrow (*Casp11*$^{-/-}$ / *Casp11*$^{-/-}$) injected with allogenic T cells from BALB/c mice is shown. Data are presented as mean ± SEM. (****$P$<0.0001; differences in animal survival were analyzed by log-rank test).

Supplementary Figure 2

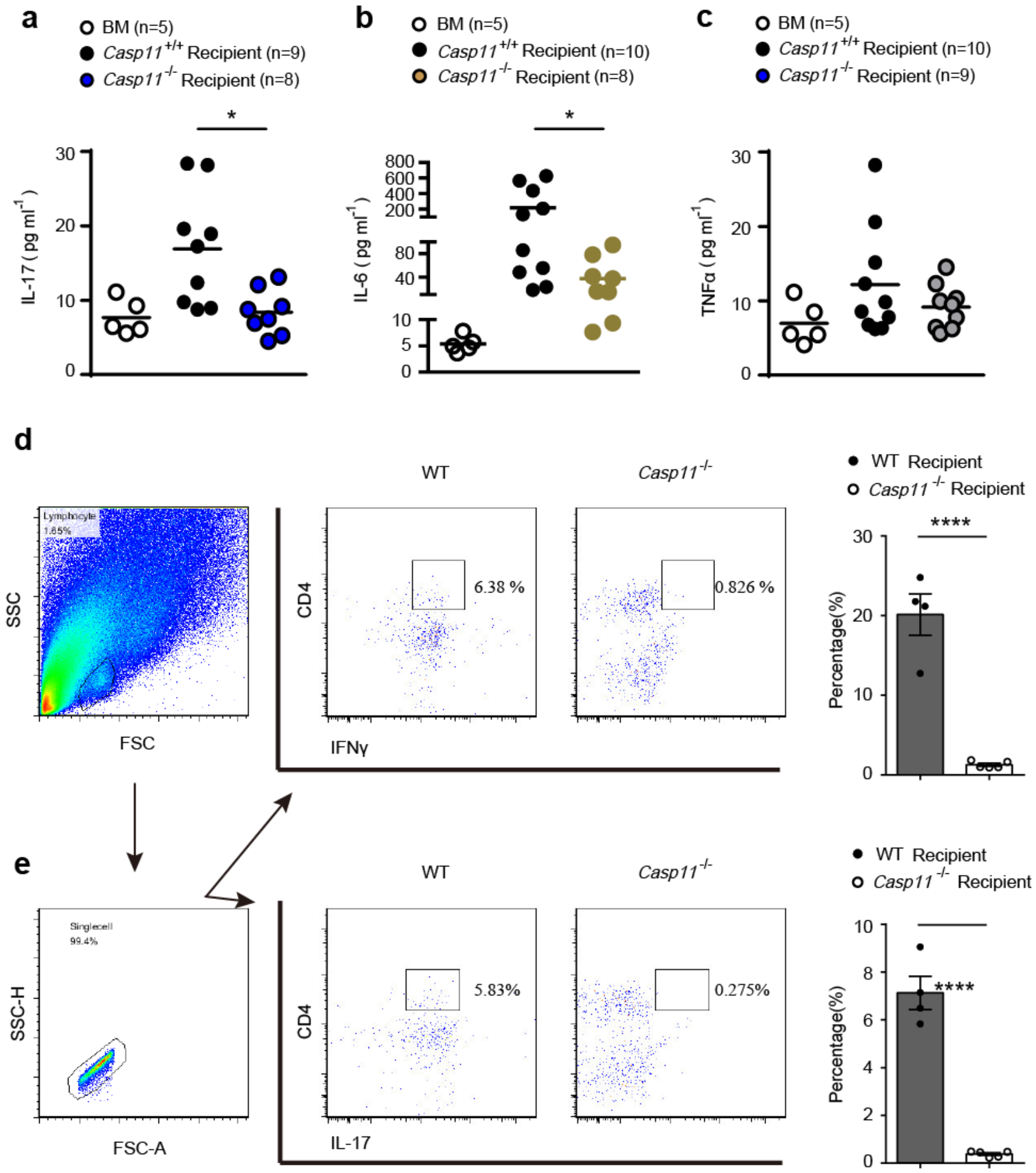


**Supplementary Figure 2: Caspase-11-deficiency attenuates Th1 and Th17 polarization. a** The IL-17 levels in the serum of the indicated groups on day 3 after allo-BMT. *$P$=0.0105, unpaired Student's $t$-test (two-sided). **b** The IL-6 levels in the serum of the indicated groups on day 3 after allo-BMT. *$P$=0.0445, unpaired Student's $t$-test (two-sided). **c** The TNF levels in the serum of the indicated groups on day 3 after allo-BMT. **d** Gating strategy to sort Th1 cells (CD45+, CD4+, IFN-γ+)

from *Casp11*$^{+/+}$ and *Casp11*$^{-/-}$ recipient mice on day 14 after TBI / BMT. Colons were isolated and digested as described in the materials and methods section. Lymphocytes from the colon underwent intracellular IFN-γstaining as described in the materials and methods section prior to analysis. Shown in the left are representative flow cytometry contour plots of frequency of intestinal Th1 cells. Shown in the right are the means ± SEM. *****P* <0.0001, unpaired Student's *t*-test (two-sided). **e** Gating strategy to sort Th17 cells (CD45+, CD4+, IL-17+) from *Casp11*$^{+/+}$ and *Casp11*$^{-/-}$ recipient mice on day 14 after TBI / BMT. Colons were isolated 14 days (n=5 for WT, n=5 for *Casp11*$^{-/-}$) and processed for single cell suspensions prior to intracellular staining. Shown in the left are representative flow cytometry contour plots of frequency of intestinal. Shown in the right are the means ± SEM. *****P* <0.0001, unpaired Student's *t*-test (two-sided).

Supplementary Figure 3

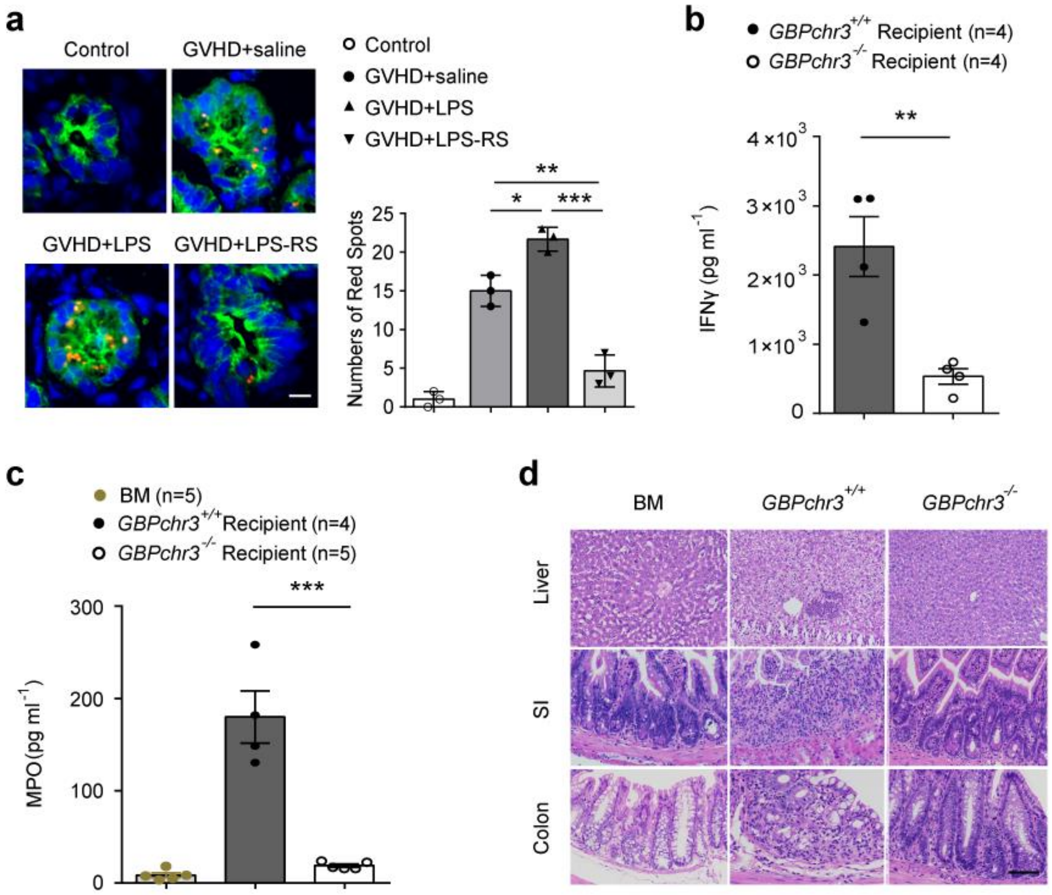


**Supplementary Figure 3: LPS-caspase-11 interaction enhances GVHD in allo-HSCT. a** The physical interaction between caspase-11 and LPS were visualized as the red spots by PLA in mouse intestinal tissue. Immunofluorescent staining was subsequently performed using anti-cytokeratin antibodies. Data are presented as mean ± SEM. GVHD+saline versus GVHD+LPS-RS, $^{**}P$=0.0034; GVHD+saline versus GVHD+LPS, $^{*}P$=0.0101; GVHD+LPS versus GVHD+LPS-RS, $^{***}P$=0.0003. Scale bar: 10 μm. **b** The IFN-γ levels in the serum of the indicated groups on day 5 after all-HSCT. The groups represent $n$ = 4 (*GBPchr*$^{+/+}$) and $n$ = 4 (*GBPchr3*$^{-/-}$) mice. Data are presented as mean ± SEM. $^{**}P$ = 0.0057, unpaired Student's $t$-test (two-sided). **c** The levels of MPO in the supernatants of the intestine in the indicated groups on day

8. The groups represent $n$ = 4 (*GBPchr3*$^{+/+}$) and $n$ =5 (*GBPchr3*$^{-/-}$) animals. Data are presented as mean ±SEM. ***$P$ = 0.0003, unpaired Student's $t$-test (two-sided). **d** Photomicrographs depicting the average disease score morphology of *GBPchr3*$^{+/+}$ and *GBPchr3*$^{-/-}$ mice are depicted. Scale bar: 100 μm.

Supplementary Figure 4

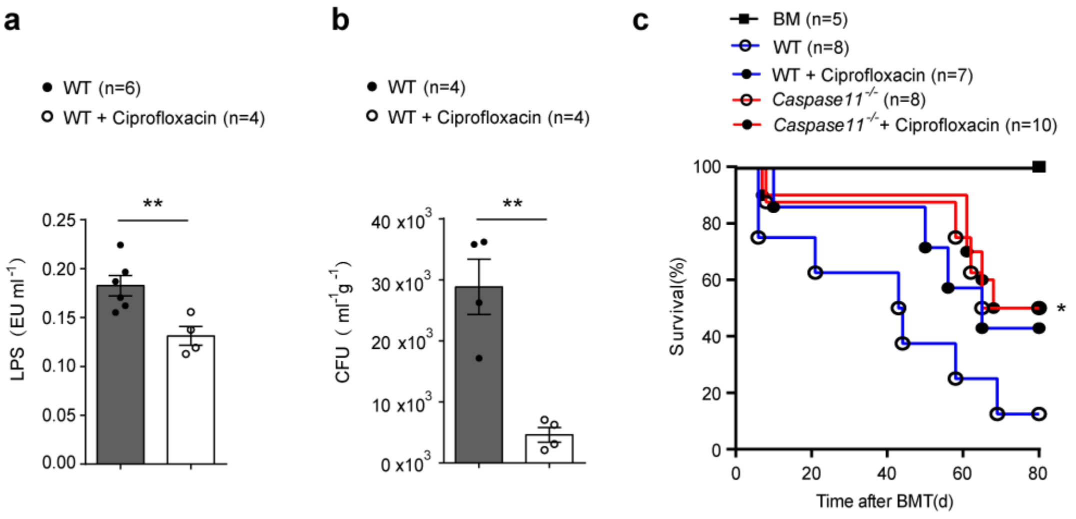


**Supplementary Figure 4: The effect of caspase-11 deficiency on GVHD reduction depends on circulating LPS or microbiota. a** The serum LPS levels are shown for mice that day 3 after TBI / BMT or TBI / BMT + Ciprofloxacin. Ciprofloxacin was given day 7 before TBI. Each data point represents an individual mouse. The mean and S.E.M. are shown. **$P$ =0.0095, unpaired Student's $t$-test (two-sided). **b** The bacterial loads in the spleen are shown for mice that day 3 after TBI / BMT or TBI / BMT + Ciprofloxacin. Ciprofloxacin was given day 7 before TBI. Each data point represents an individual mouse. The mean and S.E.M. are shown. **$P$ =0.0021, unpaired Student's $t$-test (two-sided). **c** Percentage survival of *Casp11*$^{+/+}$ and *Casp11*$^{-/-}$ recipients receiving allo-BMT from BALB/c donors after drinking ciprofloxacin water or not at the 7 d before TBI is shown. (*$P$=0.0149; differences in animal survival were analyzed by log-rank test).

Supplementary Figure 5

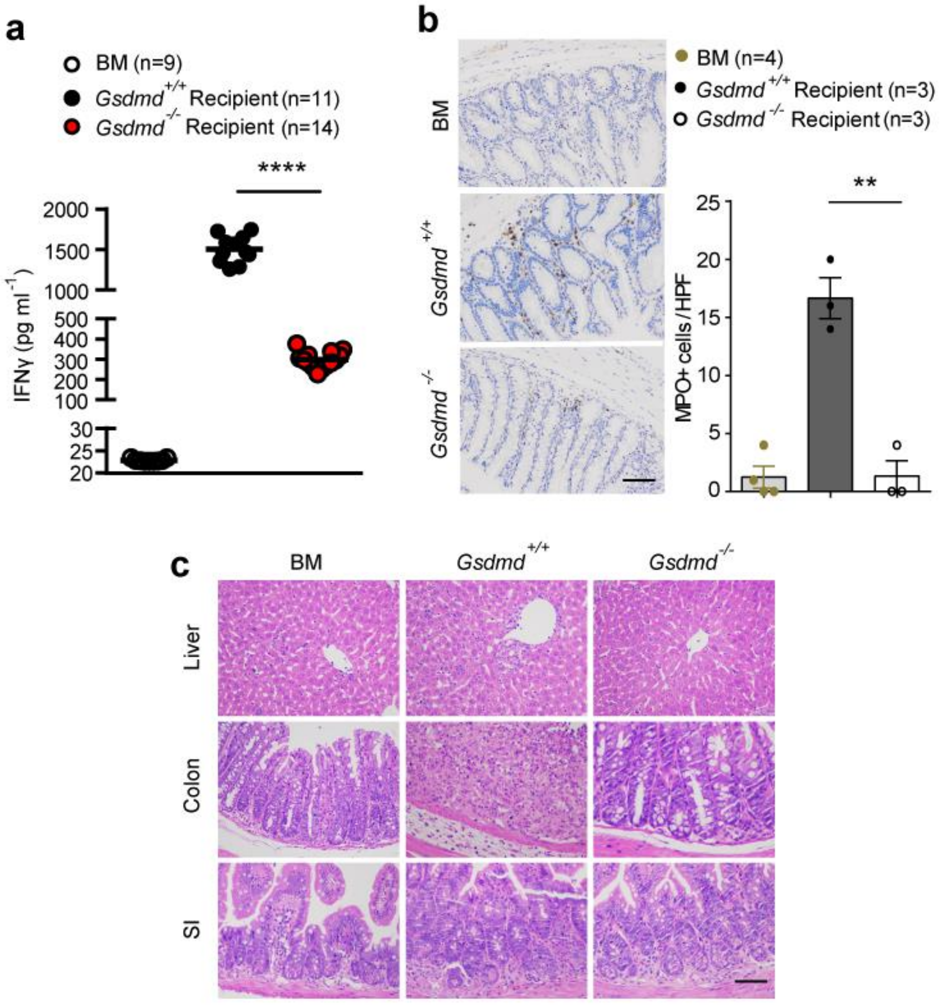


**Supplementary Figure 5: GSDMD enhances GVHD in allo-HSCT.** **a** Levels of IFN-γ in the serum of the *Gsdmd*$^{+/+}$ ( *n* = 11) and *Gsdmd*$^{-/-}$ ( *n* = 14) recipient mice on the day 5 after allo-HSCT. ****$P < 0.0001$. **b** Representative colon tissue section of *Gsdmd*$^{+/+}$ recipient mice versus *Gsdmd*$^{-/-}$ recipient mice (BALB/c→C57BL/6 combination) is shown. Staining in brown is for myeloperoxidase as indicated for the respective tissues. The frequency of myeloperoxidase positive (MPO+) cells per HPF was significantly lower in the *Gsdmd* deficient recipients than those of in WT recipients. Data (mean ±SEM) are pooled from two independent experiments. The

mean and S.E.M. are shown. **$P$ = 0.0023. Scale bar: 100 µm**.** **c** Photomicrographs depicting the average disease score morphology of *Gsdmd*$^{+/+}$ and *Gsdmd*$^{-/-}$ mice are depicted. Scale bar: 100 µm.

Supplementary Figure 6

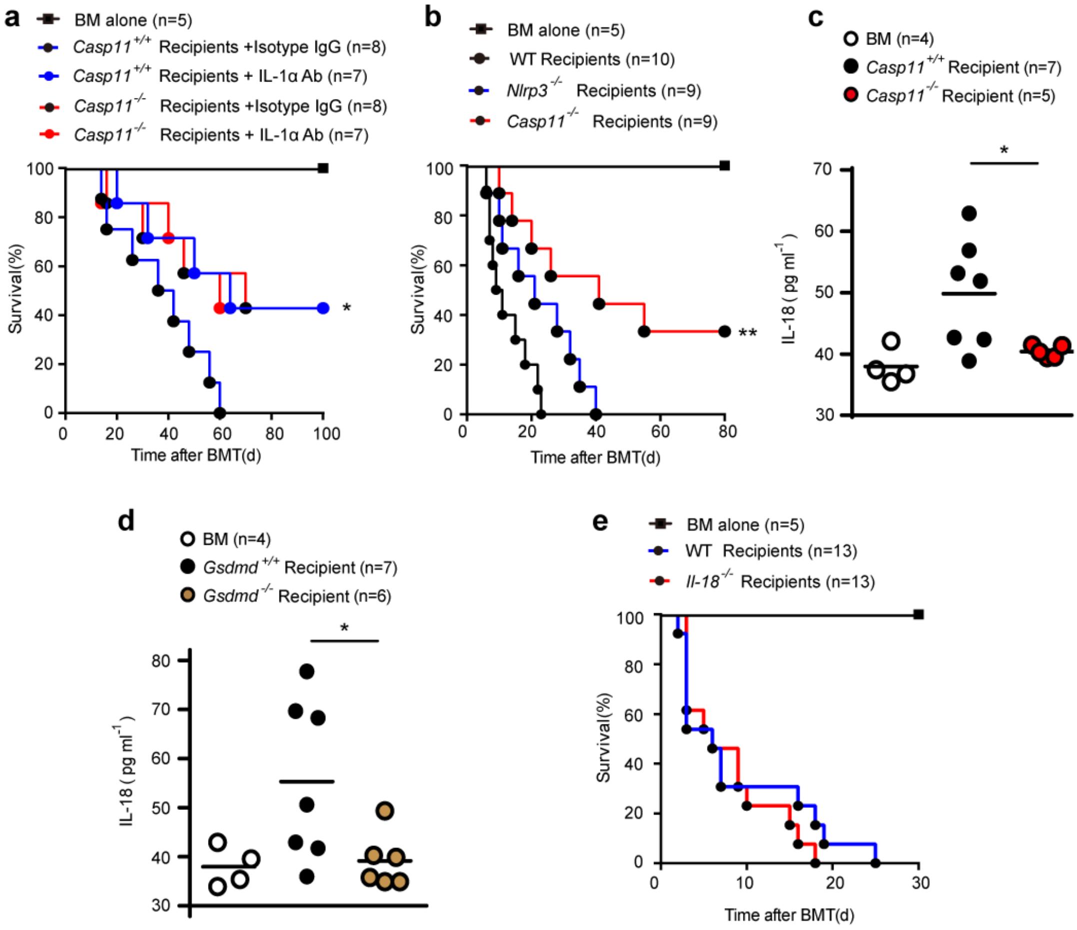


**Supplementary Figure 6: Caspase-11 signaling enhances GVHD through IL-1α but not IL-18. a** Survival of *Casp11*$^{+/+}$ and *Casp11*$^{-/-}$ recipients receiving allo-BMT from BALB/c donors injected with anti-IL-1α monoclonal antibody (4 μg/mouse) or isotype control antibody (4 μg/mouse)on day -1 and day +1 of transplantation is shown. (*$P$=0.0437; differences in animal survival were analyzed by log-rank test). **b** Percentage survival of WT, *Nlrp3*$^{-/-}$ and *Casp11*$^{-/-}$ recipient mice receiving allo-BMT with BALB/c donor cells is shown. (**$P$=0.0017; differences in animal survival were analyzed by log-rank test). **c** The IL-18 levels in the serum of the indicated groups on day 3 after allo-BMT. *$P$=0.0402, unpaired Student's $t$-test (two-sided). **d** The IL-18

levels in the serum of the indicated groups on day 3 after allo-BMT. *$P$=0.0433, unpaired Student's $t$-test (two-sided). **e** Survival of *Il-18*$^{+/+}$ versus *Il-18*$^{-/-}$ recipients receiving allo-BMT with BALB/c donor cells is shown.

Supplementary Figure 7

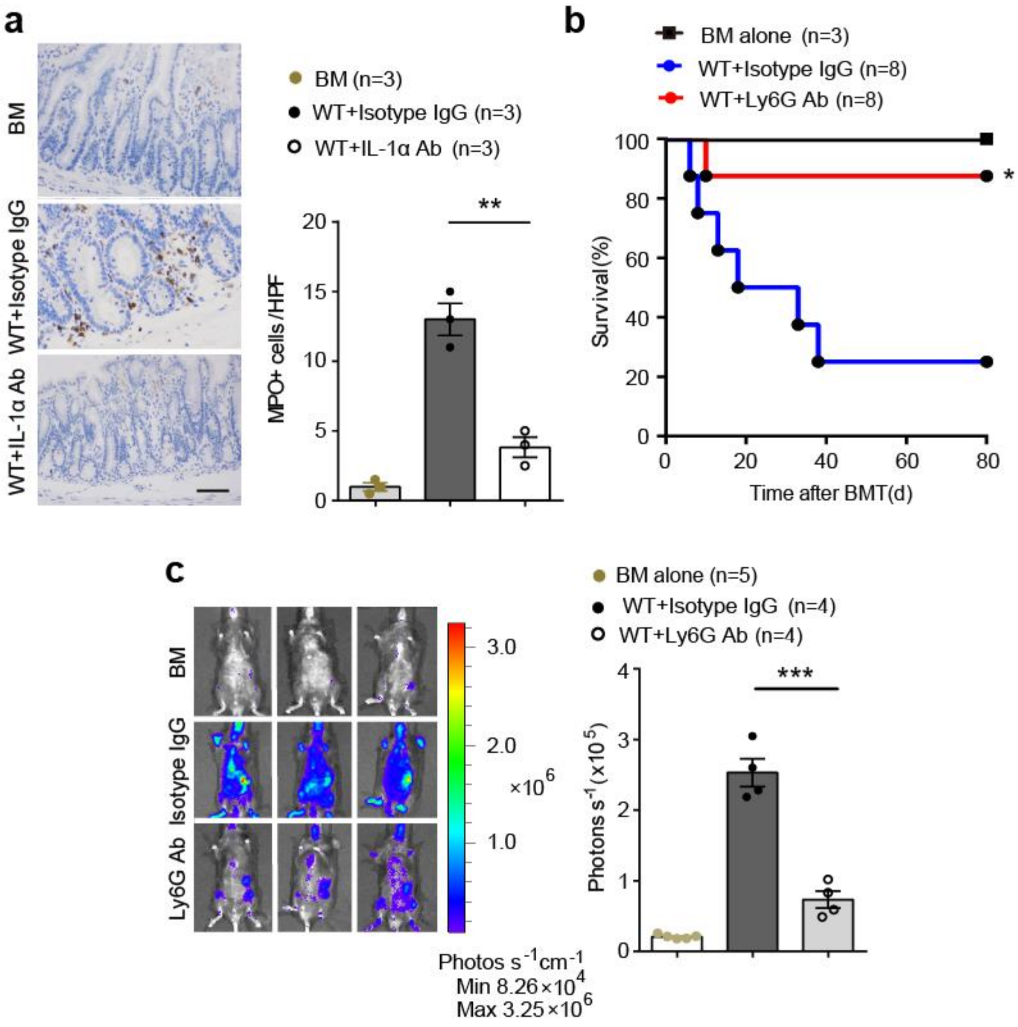


**Supplementary Figure7: IL-1 mediates neutrophil infiltration, which is critical for donor T cell expansion and GVHD-induced damage in allo-HSCT. a** Representative colon tissue sections of WT mice received BM/Tc with anti-IL-1α monoclonal antibody or isotype control antibody were shown. Staining in brown is for myeloperoxidase as indicated for the respective tissues. The frequency of myeloperoxidase positive (MPO+) cells per HPF is shown. Data are presented as mean ± SEM. ***P* =0.0026, unpaired Student's *t*-test (two-sided). Scale bar: 100 μm. **b** Percentage survival of WT mice received BM/Tc with anti-Ly6G monoclonal

antibody or isotype control antibody is shown. (**P*=0.0141; differences in animal survival were analyzed by log-rank test). **c** Forteen days posttransplant, WT mice received BM/Tc with anti-Ly6G monoclonal antibody or isotype control antibody were subjected to intraperitoneal luciferin injection and whole body BLI were depicted. Signal normalization was analyzed with Living Image software. Average representative whole body images, and average bioluminescence intensities ± SEM. ****P*=0.0002, unpaired Student's *t*-test (two-sided).

Supplementary Figure 8

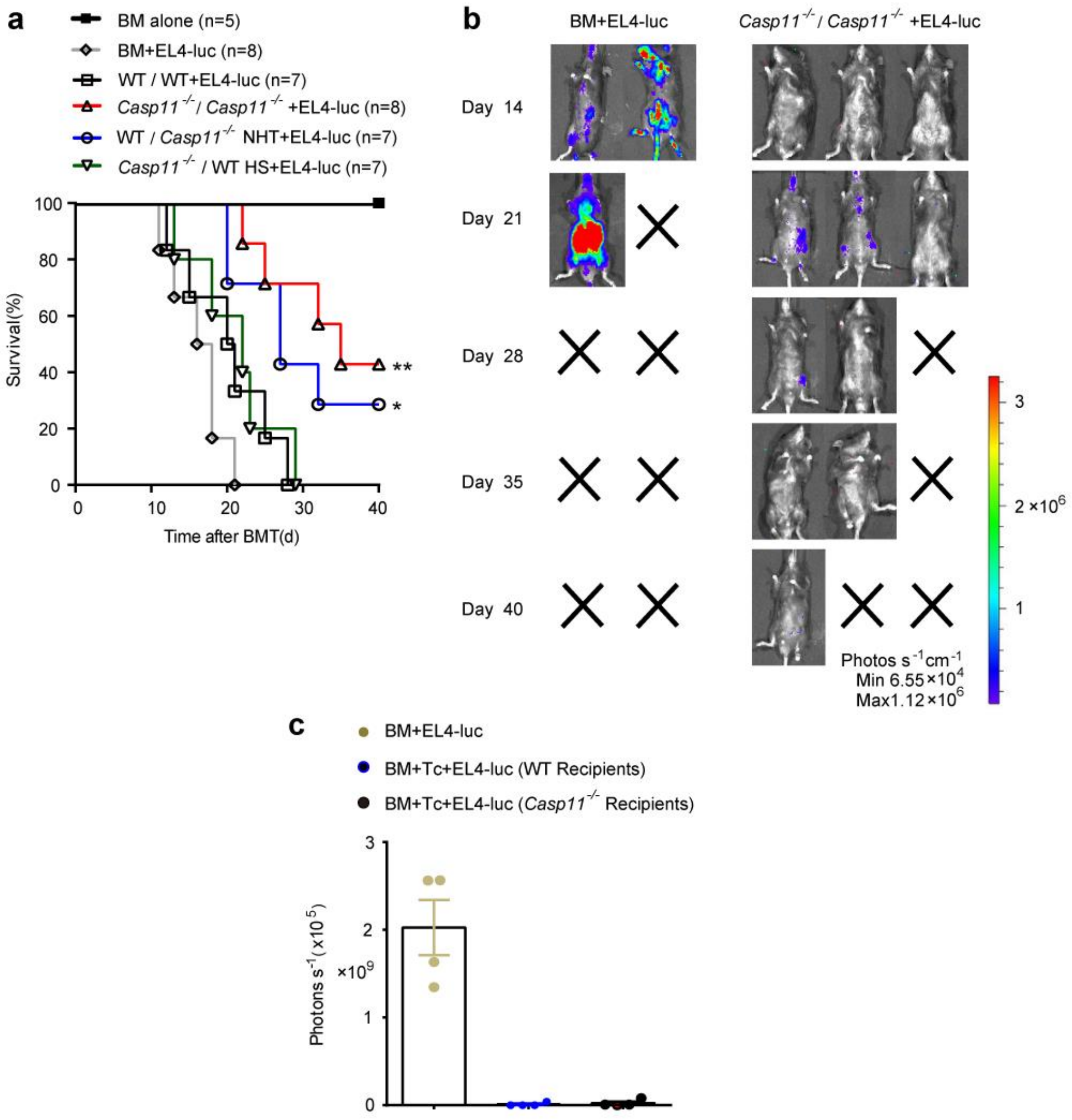


**Supplementary Figure 8: Caspase-11 deficiency preserves GVL activity. a** Percentage survival of bone marrow chimeras with *Casp11*$^{-/-}$ hematopoietic system (*Casp11*$^{-/-}$ /WT HS, the genotype left of the slash represents the hematopoietic system, the genotype right of the slash represents the non-hematopoietic compartment), or *Casp11*$^{-/-}$ non-hematopoietic compartment (WT / *Casp11*$^{-/-}$ NHT), or WT HCT mice (WT / WT), or *Casp11*$^{-/-}$ mice reconstituted with *Casp11*$^{-/-}$ bone marrow (*Casp11*$^{-/-}$ / *Casp11*$^{-/-}$) injected with allogenic T cells from BALB/c mice and EL4-luc cells is

shown. (**P*=0.0429; ***P*=0.0043, differences in animal survival were analyzed by log-rank test). **b** Tumor expansion in indicated bone marrow chimeras was shown by luciferin intraperitoneal injection and whole body BLI. **c** Tumor expansion in indicated bone marrow chimeras was shown by luciferin intraperitoneal injection and whole body BLI. Average representative whole body images, and average bioluminescence intensities ± SEM.

**Supplementary Table1: Patients Characteristics**

| Disease | Donor | GVHD grade |
|---|---|---|
| MDS | MUD | II |
| AA | Sibling | 0 |
| AML | Sibling | III |
| AML | Sibling | III |
| SAA | Sibling | II |
| AML | MUD | I |
| AML | MUD | II |
| AML | Auto | 0 |
| ALL | MRD | I |
| ALL | Sibling | III |
| ALL | MMUD | II |
| AML | MUD | III |
| MDS/MPN | MRD | III |
| SAA | Sibling | II |
| AML | Sibling | III |
| MDS | MRD | II |
| ALL | MUD | II |
| MDS | MRD | III |
| MDS | MRD | II |
| AML | MUD | 0 |
| MDS | Sibling | I |
| AML | MUD | III |
| NHL | Auto | 0 |
| CML | MRD | I |
| MM | Auto | 0 |
| AML | Sibling | II |
| ALL | Sibling | III |
| AA | Sibling | I |
| AA | Sibling | II |
| ALL | MRD | III |
| ALL | Sibling | 0 |
| AML | MUD | 0 |
| MDS | MRD | 0 |
| SAA | Sibling | III |
| ALL | Sibling | III |
| AML | MUD | III |
| MM | Auto | 0 |
| ALL | Sibling | III |

| | | |
|---|---|---|
| MM | Auto | 0 |
| ALL | Sibling | I |
| AA | MRD | 0 |
| ALL | Sibling | III |
| MDS | Sibling | II |
| AML | Sibling | IV |
| SAA | MRD | 0 |
| MDS | Sibling | III |
| ABL | Sibling | IV |
| AA | MRD | I |
| ALL | Sibling | III |
| AML | MUD | 0 |

Sibling = sibling donor, MRD = matched related donor, MUD = matched unrelated donor, MMUD = mismatched unrelated donor, Auto = Autotransplantation, AML= Acute myeloid leukemia, CML= chronic myeloid leukemia, NHL = Non Hodgkins lymphoma, ABL= Acute biphenotypic leukemia, SAA = Severe aplastic anemia, MDS= Myelodysplastic syndrome, ALL=Acute lymphoblastic leukemia, MM=Multiple myeloma.

Figure 4a. Immunoblot of GSDMD from intestine of WT, *Caspase11*$^{-/-}$ or *GBPchr3*$^{-/-}$ mice (without molecular weight markers).

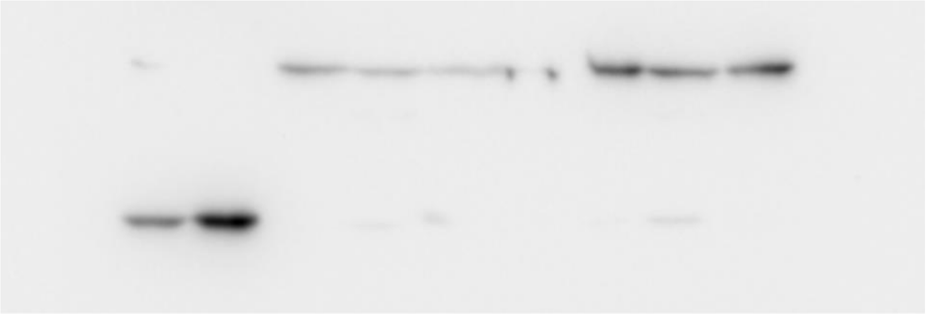

Figure 4a. Immunoblot of GSDMD from intestine of WT, *Caspase11*$^{-/-}$ or *GBPchr3*$^{-/-}$ mice (with molecular weight markers).

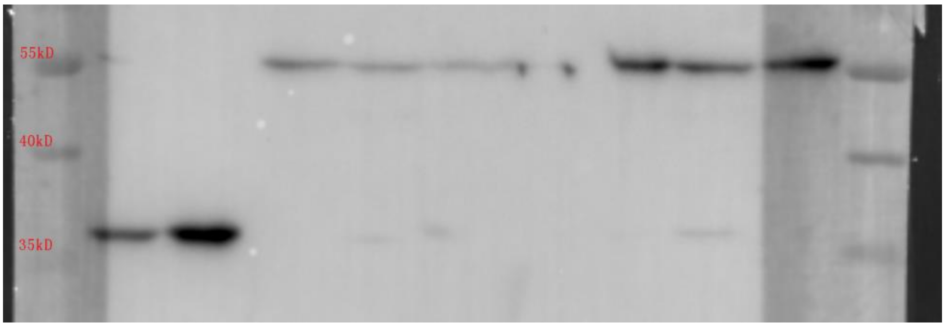


Figure 4a. Immunoblot of β-actin from intestine of WT, *Caspase11*$^{-/-}$ or *GBPchr3*$^{-/-}$ mice (with molecular weight markers).

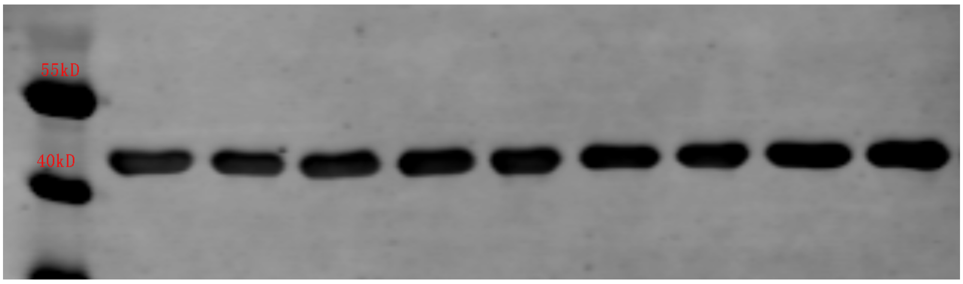


Figure 4a. Immunoblot of GSDMD from intestine of WT, *Caspase11*$^{-/-}$ or *Gsdmd*$^{-/-}$ (without molecular weight markers).

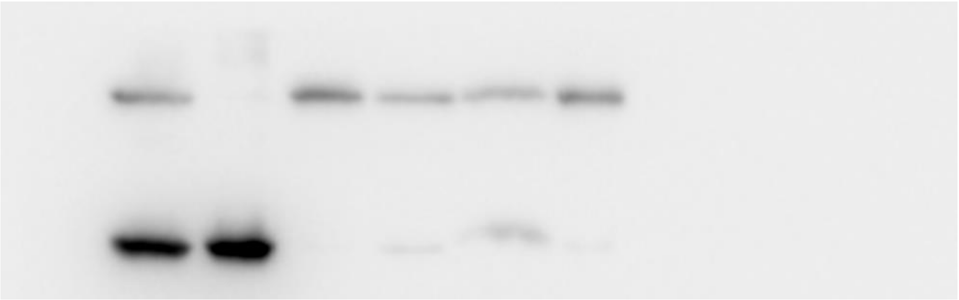

Figure 4a. Immunoblot of GSDMD from intestine of WT, *Caspase11*$^{-/-}$ or *Gsdmd*$^{-/-}$ (with molecular weight markers).

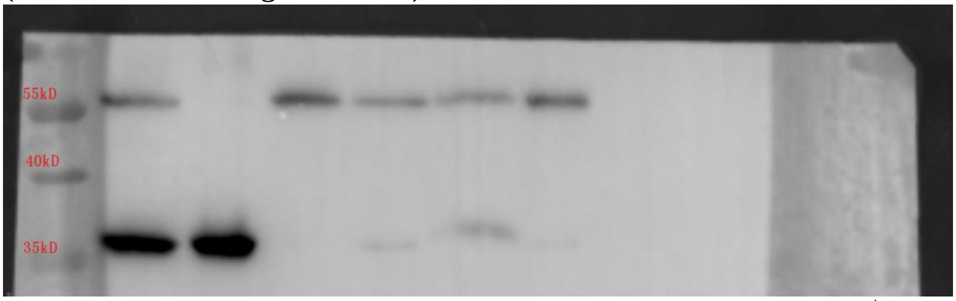


Figure 4a. Immunoblot of β-actin from intestine of WT, *Caspase11*$^{-/-}$ or *Gsdmd*$^{-/-}$ (with molecular weight markers).

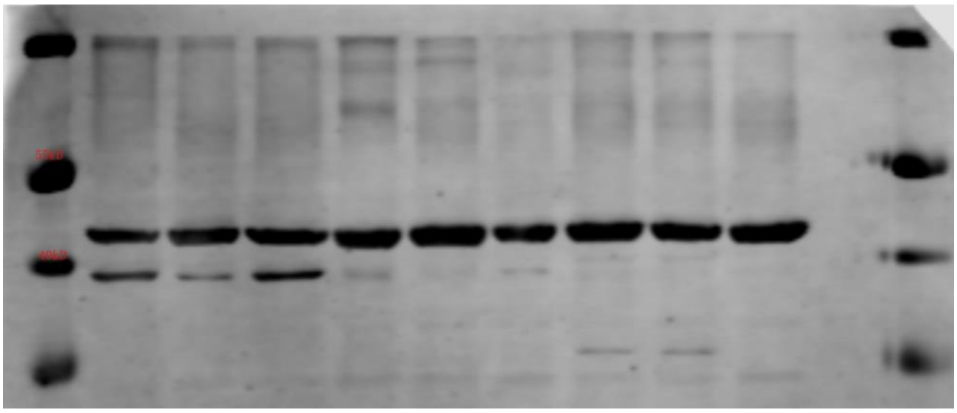

Supplement: Supplementary file 1 — Supplementary Information [file 41467_2019_11895_MOESM1_ESM.pdf]
